# The ortholog of human DNAJC9 promotes histone H3–H4 degradation and is counteracted by Asf1 in fission yeast

**DOI:** 10.1093/nar/gkaf036

**Published:** 2025-01-29

**Authors:** Yan Ding, Jun Li, He-Li Jiang, Fang Suo, Guang-Can Shao, Xiao-Ran Zhang, Meng-Qiu Dong, Chao-Pei Liu, Rui-Ming Xu, Li-Lin Du

**Affiliations:** College of Life Sciences, Beijing Normal University, Beijing 100875, China; National Institute of Biological Sciences, Beijing 102206, China; National Institute of Biological Sciences, Beijing 102206, China; Key Laboratory of Epigenetic Regulation and Intervention, Institute of Biophysics, Chinese Academy of Sciences, Beijing 100101, China; National Institute of Biological Sciences, Beijing 102206, China; National Institute of Biological Sciences, Beijing 102206, China; National Institute of Biological Sciences, Beijing 102206, China; National Institute of Biological Sciences, Beijing 102206, China; Tsinghua Institute of Multidisciplinary Biomedical Research, Tsinghua University, Beijing 102206, China; Key Laboratory of Epigenetic Regulation and Intervention, Institute of Biophysics, Chinese Academy of Sciences, Beijing 100101, China; Key Laboratory of Epigenetic Regulation and Intervention, Institute of Biophysics, Chinese Academy of Sciences, Beijing 100101, China; College of Life Sciences, Beijing Normal University, Beijing 100875, China; National Institute of Biological Sciences, Beijing 102206, China; Tsinghua Institute of Multidisciplinary Biomedical Research, Tsinghua University, Beijing 102206, China

## Abstract

Mammalian J-domain protein DNAJC9 interacts with histones H3–H4 and is important for cell proliferation. However, its exact function remains unclear. Here, we show that, in the fission yeast *Schizosaccharomyces pombe*, loss of Djc9, the ortholog of DNAJC9, renders the histone chaperone Asf1 no longer essential for growth. Utilizing AlphaFold-based structural prediction, we identified a histone-binding surface on Djc9 that binds to helix α3 of H3 in a manner that precludes simultaneous helix α3-binding by Asf1. Djc9 and Asf1 indeed compete for binding to the H3–H4 dimer *in vitro*, and an H3-α3 mutation impeding Djc9 binding also renders Asf1 non-essential, indicating that the role of Asf1 needed for growth in fission yeast is to prevent histone binding by Djc9. In the absence of Asf1, cell growth is hindered due to unrestrained Djc9-mediated downregulation of H3 and H4. In the presence of Asf1, Djc9 confers resistance to the DNA replication inhibitor hydroxyurea and dominant negative disease-related histone mutants by promoting the degradation of superfluous or dysfunctional histones. Our findings provide new insights into the function and mechanism of this conserved histone-binding protein.

## Graphical Abstract

**Graphical Abstract:**
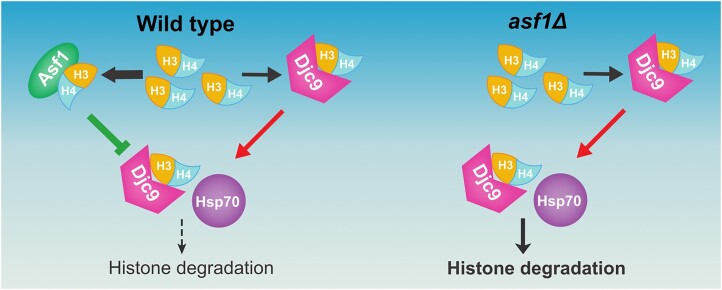

## Introduction

In eukaryotes, canonical histone proteins play a pivotal role in compacting genomic DNA into nucleosomes, which constitute the fundamental units of chromatin. Each nucleosome consists of ∼146 bp of DNA wound about 1.6 turns around an octamer, comprising one histone (H3–H4)_2_ tetramer and two H2A–H2B dimers [1]. During the S phase of the cell cycle, a large amount of new histones must be synthesized to allow the assembly of newly replicated DNA into nucleosomes. Histone chaperones, a diverse group of proteins that bind to histones, are required for the proper assembly of newly synthesized histones into chromatin [2, 3].

Asf1 is a highly conserved histone chaperone and binds to the H3–H4 dimer, shielding the surface involved in forming the (H3–H4)_2_ tetramer [4, 5]. In the budding yeast *Saccharomyces cerevisiae*, Asf1 promotes the acetylation of H3 at lysine 56 (H3K56ac), which marks newly synthesized H3 and regulates nucleosome assembly, transcription, and DNA repair [6–8]. H3K56ac also exists in the fission yeast *Schizosaccharomyces pombe* [9, 10]. However, whether Asf1 contributes to histone H3K56 acetylation in *S. pombe* has not been assessed. Asf1 is dispensable for cell growth in *S. cerevisiae* [11]. In contrast, Asf1 is essential for cell growth in *S. pombe* [12]. The reason behind this difference in essentiality is currently unknown.

Histone levels need to be tightly controlled. Insufficiency of histone supply during S phase causes cell cycle arrest [13, 14]. Conversely, an excess of histones carries deleterious consequences, rendering cells sensitive to DNA damage and increasing genome instability [15, 16]. During a normal cell cycle, histone synthesis is intricately synchronized with DNA replication [17, 18]. However, exposure to genotoxic agents that interfere with DNA replication may disrupt the delicate balance between histone synthesis and DNA synthesis, resulting in an accumulation of excess non-nucleosomal histones during S phase [19–21, 22]. Multiple mechanisms exist in eukaryotic cells to downregulate histone mRNA levels in response to genotoxic stresses [23–25].

Cells also regulate histone abundance at the protein level. In *S. cerevisiae*, the checkpoint kinase Rad53 promotes the ubiquitination-dependent degradation of overexpressed histones [21, 26], and the protease Wss1 cleaves ssDNA-bound histones that accumulate when cells are challenged with the DNA replication inhibitor hydroxyurea (HU) [16, 26, 27]. It is unclear whether and how these histone degradation mechanisms are counteracted to prevent unwanted histone degradation during unperturbed S phase.

J-domain proteins are co-chaperones of HSP70 and are involved in targeting HSP70 to specific substrate proteins [28, 29]. In mammalian cells, a J-domain protein DNAJC9 was found to co-purify with histones H3 and H4 more than a decade ago [20,30, 31]. Only recently have its roles in histone regulation begun to be understood [32–34]. One study proposed that DNAJC9, through recruiting HSP70 to safeguard the structural integrity of histones, ensures sufficient histone supply and facilitates histone deposition onto chromatin [32]. Another group proposed that DNAJC9 is functionally linked to ASF1, based on an intriguing negative correlation observed in the essentiality profiles of DNAJC9 and ASF1; however, the exact nature of their relationship remains unclear [33]. These findings invite further exploration of the roles of DNAJC9 in histone homeostasis.

Here, we show that the *S. pombe* ortholog of the human DNAJC9, Djc9, acts with Hsp70 to promote the degradation of histones H3 and H4 by the proteasome. During the unperturbed cell cycle, Djc9 is inhibited by Asf1 via competitive binding to the H3–H4 dimer. In the absence of Asf1, excessive Djc9-dependent degradation of histones H3 and H4 causes lethality. In the presence of Asf1, Djc9 confers cellular tolerance to HU and protects cells from toxicity caused by dominant-negative histone mutants. Our findings shed new light on the roles of this conserved histone-binding protein.

## Material and methods

### Fission yeast strain and plasmid construction

Fission yeast strains used in this study are listed in Supplementary Table S1, and plasmids used in this study are listed in Supplementary Table S2. Genetic methods and recipes for yeast culture medium are as described [35]. Gene deletion strains used in this study were generated by polymerase chain reaction (PCR)-based gene targeting [36, 37]. Strains expressing C-terminal endogenously tagged Ssa1 (Ssa1-GFP), Djc9 (Djc9-TAP), and Asf1 (Asf1-13 × Myc) were constructed using PCR-based gene tagging [38].

The *asf1-AID* strain was constructed using an improved AID system, with some details differing from the published protocol [39]. Using PCR-based endogenous tagging, a 3 × sAID degron was fused to the C-terminus of Asf1, and a spo5DSR element that reduces the mRNA level was added downstream of the coding sequence [40]. The *Arabidopsis thaliana* F-box protein TIR1 (AtTIR1), harboring F79A/D170E/M473L mutations, was expressed under the *Padh1* promoter from a stable integrating vector (SIV) pAde6^PmeI^-hphMX (pAV0585) [41]. The degradation of Asf1-AID was induced with 0.2 μM of Ad-IAA (TCI, A3390).

The *PtetO7-djc9* strain was constructed as previously described [42]. Briefly, the *PtetO7-TATACYC1* cassette from the plasmid pFA6a-hphMX-tetO-Pcyc1-3xFLAG (Addgene, 41 020) was inserted upstream of the start codon of *djc9* by PCR-based targeting. The plasmid pDM291-tetR-tup11Δ70 (Addgene, 41 027) was linearized with BamHI and integrated at the *ura4* locus.

For the expression of human DNAJC9 in *S. pombe*, we cloned the cDNA of human DNAJC9 into an integration plasmid containing the inducible *PenotetSW2* promoter [43]. The expression of DNAJC9 was induced with 2.5 μg/mL of anhydrotetracycline (ahTet) (Sigma Aldrich, 37 919).

For phenotype analysis, the wild-type Djc9 or a Djc9 mutant tagged with mCherry at the C-terminus was expressed under the native promoter from a pAde6^PmeI^-hphMX-based SIV plasmid integrated at the *ade6* locus. To examine the interaction with Ssa1-GFP, the wild-type Djc9 or HPD motif-mutated Djc9 under the *Pnmt41* promoter was expressed from a plasmid based on a modified SIV vector pAde6^NotI^-hphMX. The wild-type Asf1 and Asf1 mutants with a C-terminal YFP-FLAG-His_6_ (YFH) tag were expressed under the native promoter from a pDUAL vector-based plasmid integrated at the *leu1* locus [44].

Plasmids expressing H3–H4 were constructed by cloning the divergently transcribed gene pair of *hht2-hhf2* (spanning from 323 bp downstream of *hhf2* to 330 bp downstream of *hht2*) into a pDUAL vector. For *Pinv1*-H3-FLAG plasmid construction, the promoter region of *inv1* (SPCC191.11) was amplified with the forward primer 5′-agcacgacttttgatccgttcg-3′ and the reverse primer 5′-gcaaatcttcaaagttagcg-3′, and placed upstream of the coding sequence of H3-FLAG in the modified SIV vector pAde6^NotI^-hphMX or the pDUAL vector. Plasmids for screening dominant-negative forms of histones in *djc9Δ* cells were constructed by cloning the histone coding sequence upstream of a FLAG tag and downstream of the *Pnmt1* promoter in a pDUAL vector.

Tetrad dissection analysis was performed as previously described [45].

### Growth phenotype analysis (spot assay)

For the drug sensitivity test, log-phase cells grown in YES medium were washed twice with water and diluted to an OD_600_ of 0.4 in water. Five-fold serial dilutions of cells were performed in a 96-well plate and spotted on a plate without the drug (no treatment control), or plates containing drugs at different concentrations using a replica plater (Sigma, R2383). Plates were incubated in the incubator at 30°C and scanned 2 days later. To analyze the growth phenotype of *asf1* ts mutants, strains were spotted on YES plates and incubated at a permissive temperature (25°C) and a restrictive temperature (30°C or 33°C). To analyze the toxicity of H3-GFP or H3 mutants expressed from the thiamine-repressible *Pnmt1* promoter, cells grown in Edinburgh minimal medium (EMM) containing 5 μg/mL of thiamine were washed three times with water and spotted on EMM plates with or without thiamine to repress or induce expression, respectively.

### Protein extraction and immunoblotting analysis

Whole-cell protein extracts were prepared as previously described [46]. Briefly, 3 OD_600_ units of cells were lysed using 1.85 M NaOH and 7.5% (v/v) β-mercaptoethanol and proteins were precipitated by adding an equal volume of 55% trichloroacetic acid (TCA, Sigma Aldrich). After centrifugation, pellets were washed twice with ice-cold acetone and dissolved in the HU sample buffer (8 M urea, 200 mM Tris-HCl, pH 6.8, 1 mM EDTA, 5% sodium dodecyl sulfate (SDS), 0.1% bromophenol blue, 10 mM dithiothreitol). Protein samples were denatured at 95°C for 5 min, then separated by 4–20% gradient SDS-polyacrylamide gel electrophoresis (SDS-PAGE) and immunoblotted.

The antibodies used for immunoblotting were: a rat polyclonal antibody against Djc9 and a rat polyclonal antibody against Asf1 generated in this study; rabbit polyclonal anti-H2A antibody (GeneTeX, GTX129418), rabbit polyclonal anti-H2B antibody (GeneTeX, GTX129565), rabbit polyclonal anti-H3 antibody (Abcam, ab1791); rabbit polyclonal anti-H4 antibody (Abcam, ab10158); rabbit polyclonal anti-H3K56ac antibody (Sigma, 07–677-IS); mouse monoclonal anti-mini-AID tag antibody (MBL, M214-3); mouse monoclonal anti-FLAG M2 antibody (Sigma, F1804); mouse monoclonal anti-GFP antibody (Roche, 11 814 460 001); mouse monoclonal anti-c-Myc antibody (Roche, 11 667 203 001); monoclonal anti-GST-HRP (Sigma, A7304); peroxidase anti-peroxidase (PAP) antibody for detecting TAP-tagged proteins (Sigma, P1291); rabbit polyclonal anti-Lap2 antibody (Du lab stock).

### Immunoprecipitation

About 100 OD_600_ units of cells were collected and washed twice with water. The cell pellets were mixed with 200 μL of lysis buffer (6 mM Na2HPO4, 4 mM NaH2PO4, 150 mM NaCl, 2 mM EDTA, 10% glycerol, 1 mM DTT, 1 mM PMSF, 0.05% NP40, 1 × protease inhibitor cocktail (Roche)) and 800 μL of 0.5 mm diameter glass beads. Bead-beating cell lysis was performed using a FastPrep-24 instrument at a setting of 6.5 m/s for 20 s, repeating three times with a 5-min interval on ice. After bead beating, cell lysates were separated from glass beads and another 800 μL of lysis buffer was added to each sample. Then the cell lysates were centrifuged at 15 000 × *g* for 30 min at 4°C. The supernatants were incubated with GFP-Trap agarose (Chromo Tek, gta-20) for the immunoprecipitation (IP) of GFP-tagged protein, anti-FLAG M2 agarose (Sigma, A2220) for the IP of FLAG-tagged protein, IgG Sepharose (GE healthcare, 17–0969-01) for the IP of TAP-tagged protein. After a 3-h incubation at 4°C, antibody beads were washed six times with the lysis buffer and beads-bound proteins were eluted with the HU sample buffer for the IP of GFP or TAP-tagged proteins, or 3 × FLAG peptide (Sigma, F4799) for the IP of FLAG-tagged proteins.

### Recombinant protein expression and purification

For the *in vitro* competition binding assay, full-length Djc9 and Asf1 were expressed as N-terminal GST fusion proteins in the BL21 (DE3) strain of *Escherichia coli* using a plasmid vector modified from pETDuet-1 (Novagen, 71 146). *E. coli* was first cultured at 37°C in LB medium supplied with 50 μg/mL of ampicillin until the cell density reached an OD_600_ unit of 0.6–0.8. Then, expression was induced by 0.5 mM IPTG at 16°C for 16–20 h. Cells were harvested by centrifugation and resuspended in lysis buffer (20 mM Tris-HCl, pH 8.0, 300 mM NaCl, 5% glycerol, 1 mM DTT, 1 mM PMSF, 1 × protease inhibitor cocktail), then lysed by sonication. Lysates were cleared by centrifugation and supernatants were incubated with glutathione Sepharose beads (GE Healthcare, 17–0756-01). After 2 h of incubation at 4°C, the beads were washed 4 times using lysis buffer plus 0.2% (v/v) Triton X-100, and HRV 3C protease was used to cleave between the GST tag and the recombinant protein in the cleavage reaction buffer (50 mM Tris-HCl, pH 7.0, 300 mM NaCl, 1 mM EDTA, 1 mM DTT).

Fission yeast histones H3 and H4 were co-expressed using a pCDFDuet-1-based plasmid in the BL21 (DE3) strain of *E. coli* for 4 h at 37°C with 0.5 mM IPTG. Cells were sonicated in histone lysis buffer A (20 mM Tris-HCl, pH 7.5, 500 mM NaCl, 5% glycerol, 1 mM EDTA, 1 mM DTT). Histones were first purified by heparin resins (GE healthcare) and eluted with a 0.5–2 M NaCl gradient in histone lysis buffer. The eluted fractions were collected and analyzed by Coomassie blue-stained SDS-PAGE, and the first two peaks enriched with histones H3 and H4 were chosen for further purification through a HiLoad 16/600 Superdex 200 gel filtration column (GE healthcare) in gel filtration buffer (20 mM Tris-HCl, pH 7.5, 2 M NaCl, 5% glycerol, 1 mM EDTA, 1 mM DTT). Fractions containing histones H3 and H4 were pooled and concentrated to ∼5 mg/mL and stored at −80°C.

### *In vitro* GST-Djc9 pull-down assay

To examine the interaction between Djc9 and histones H3 and H4, GST-tagged Djc9 was expressed in the BL21 (DE3) strain of *E. coli* using a pGEX4T-1 vector (GE Healthcare). The protein was purified following the same protocol used for Djc9 expressed from the pETDuet-1-based plasmid, except that no cleavage was performed so that GST-Djc9 remained bound to the glutathione Sepharose beads. The GST-Djc9 pull-down assay was carried out by mixing 5 μg of glutathione beads-bound GST-Djc9 with 5 μg of purified histones H3 and H4 in 200 μL of binding buffer (1 × phosphate-buffered saline (PBS), 1 M NaCl, 1 mM DTT, 10% glycerol, 1 × protease inhibitor cocktail) and incubated for 1 h at 4°C. After incubation, the beads were washed four times with binding buffer plus 0.2% (v/v) Triton X-100 before eluting using 50 μL of the HU sample buffer. Then all the samples were analyzed by Coomassie blue-stained SDS-PAGE.

### *In vitro* competition assay between Djc9 and Asf1 for H3–H4 binding

Around 5 μg of glutathione beads-bound GST-Djc9 or GST-Asf1 were mixed with 5 μg of purified histones H3 and H4 in the presence of purified Asf1 or Djc9 at concentrations of 0, 2.5, 5, and 10 μg in 200 μL of binding buffer (1 × PBS, 1 M NaCl, 1 mM DTT, 10% glycerol, 1 × protease inhibitor cocktail). After 1 h of incubation at 4°C, the beads were washed four times with binding buffer plus 0.2% (v/v) Triton X-100. Samples were analyzed by Coomassie blue-stained SDS-PAGE.

### RT-qPCR analysis of histone H3 transcript

Total RNA was isolated from 5 OD_600_ units of cells using a standard RNA extraction protocol [47]. For cDNA synthesis, 1 μg of total RNA from each sample was subjected to a reverse transcription reaction with oligo(dT)_12_ and random primers (Takara, RR037A). The resulting reverse transcription reaction was diluted at a 1:10 ratio, and 2 μL of the diluted reaction was used as the cDNA template for qPCR analysis on a Bio-Rad CFX96 real-time PCR system. The expression levels of the H3-FLAG transcripts were normalized to those of actin transcripts. H3-FLAG transcripts were amplified using the forward primer 5′- ctatccatggcaagcgtgtta -3′ and the reverse primer 5′- tcgtcgtccttgtagtcagag -3′. Primers for amplifying actin transcripts were sourced from a previously published study [48].

### *In vitro* Ni-NTA pull-down assay

To assess the interaction between histones H3–H4 and different truncated forms of Djc9, a pCDFDuet-1-based plasmid expressing H3-FLAG_2_-His_6_ (H3-FFH) and H4 was co-transformed with a pGEX4T-1-based plasmid expressing GST-Djc9 or a GST-fused truncated fragment of Djc9 into *E. coli* strain BL21 (DE3). Plasmids were selected using 50 μg/mL of streptomycin for the pCDFDuet-1-based plasmid and 50 μg/mL of ampicillin for the pGEX4T-1-based plasmid. Protein expression was induced with 0.5 mM IPTG at 16°C for 16–20 h. About 50 OD_600_ units of cells for each sample were collected and lysed by bead beating as performed for IP in lysis buffer (20 mM Tris-HCl, pH 7.5, 500 mM NaCl, 5% glycerol, 1 mM EDTA, 1 mM DTT, 10 mM imidazole, 1 mM PMSF, 1 × protease inhibitor cocktail). Protein purification was performed using Ni-NTA agarose beads at 4°C for 2 h. Then, the beads were washed four times with lysis buffer plus 0.2% (v/v) Triton X-100, and the bound proteins were eluted with 250 mM imidazole in lysis buffer. The bound proteins were analyzed by immunoblotting.

### AlphaFold-multimer prediction

Structures of the Djc9–H3–H4 complex, the human DNAJC9–H3.3–H4 complex, and the Asf1–H3–H4 complex were predicted using AlphaFold version 2.3.0. The software was downloaded from GitHub and installed following the instructions provided at https://github.com/google-deepmind/alphafold. We set the –model_preset option to ‘multimer’ to run AlphaFold-Multimer. The –db_preset option was set to ‘full_dbs’ to utilize the complete databases for multiple sequence alignments (MSAs). By default, AlphaFold-Multimer generated a total of 25 predicted structures (five seeds per model × five models). These predicted structures were ranked according to the model confidence score, which is a weighted combination of pTM and ipTM (0.2 × pTM + 0.8 × ipTM) as described in [49]. The top-ranked structure with the highest confidence score was selected for further analysis.

The structures were visualized and analyzed using PyMOL (version 2.5.2). The interface area was calculated using the PDBPisa web server (https://www.ebi.ac.uk/pdbe/pisa/) [50].

### Screening for histone mutants suppressing the growth defect of *asf1-30* using error-prone rolling circle amplification

To screen histone mutants that could suppress the growth defect of *asf1-30* cells at a restrictive temperature, rolling circle amplification (RCA) was used to introduce mutations on the *leu1*-marked plasmid pDUAL*-hht2–FLAG-hhf2* (FLAG tagged at the N terminus of *hhf2*) according to a previously published procedure [51] with some modifications. Briefly, 1 ng of pDUAL*-hht2–FLAG-hhf2* plasmid was added to 10 μL of mutagenesis reaction (1 × phi29 DNA polymerase reaction buffer, 25 μM random primer, 2 mM MnCl2, 0.5 mM dNTP, 0.2 mg/mL BSA, 5 units of phi29 DNA polymerase). A total of 20 reactions were performed in parallel. After 24 h of incubation at 30°C, the RCA reaction products were digested with DpnI to remove the template, linearized with NotI, and purified using the QIAquick PCR Purification Kit (QIAGEN) to exchange the ionic buffer with ion-free water. The purified DNA was transformed into *asf1-30* cells using an electroporator (Gene Pulser Xcell Electroporation System, PC module) following a previously published protocol [52]. The transformed cells were plated on leucine-free EMM plates and recovered at 25°C for 24 h and then incubated at 37°C to select the suppressor-containing clones, using *asf1-30* cells transformed with unmutagenized pDUAL*-hht2–FLAG-hhf2* as a negative control. Colonies that emerged on plates containing cells transformed with RCA mutagenized plasmids were picked up and further verified by spot assay. Genomic DNA was then extracted from the verified clones. Primers annealing to the FLAG sequence or to a vector backbone sequence were used to specifically amplify the DNA sequences of *hht2* and *hhf2* from the integrated plasmid. The amplified PCR products were sequenced by Sanger sequencing.

### Screening for dominant-negative histone mutant toxic to *djc9Δ* cells but not wild-type cells

Loss-of-function histone mutations were chosen based on a published analysis of histone mutations [53]. A total of 32 lethal mutations of histone H3 and 20 lethal mutations of histone H4 were selected for examination. A pDUAL-based plasmid expressing wild-type or mutant histone under the *Pnmt1* promoter was transformed into *djc9Δ* cells and wild-type cells. Equal amounts of the transformation reaction were plated on a thiamine-containing EMM plate and a thiamine-free EMM plate. The plates were incubated at 30°C for 4 days. The number and size of transformant colonies on thiamine-containing and thiamine-free plates were compared.

### Flow cytometry analysis

Flow cytometry analysis was performed as previously described [54]. Briefly, about 0.2 OD_600_ units of cells were fixed with 70% pre-chilled ethanol and then incubated with 1 μM Sytox Green (Invitrogen) in 50 mM sodium citrate containing 0.1 mg/mL RNase (Sigma) overnight. Immediately before analysis, the cells were briefly sonicated. Samples were analyzed using a BD FACSAria II instrument (BD Biosciences), and the data were processed using the FlowJo v10.8.1 software (BD Biosciences).

### Microscopy

Live-cell imaging was performed as previously described [55].

### Phylogenetic analysis of Djc9 orthologs

For the analysis of the conservation and divergence of DNAJC9 homologs across eukaryotes, sequences containing the PANTHER domain ‘DNAJ HOMOLOG SUBFAMILY C MEMBER 9″ (PTHR44144) were retrieved from the PANTHER database [56]. The MSA was performed using the MAFFT E-INS-i algorithm [57]. The MSA was further visualized and adjusted using Jalview v2 [58] to remove the highly divergent N-terminal region and C-terminal regions of the orthologs. Finally, DNAJC9 homologs from 9 fungal species, 14 metazoan species, and 4 non-Opisthokonta species were selected for the maximum likelihood phylogenetic tree calculation using IQ-TREE 2 [59]. The tree was rooted using proteins from the four non-Opisthokonta species as outgroup and visualized using FigTree (v.1.4.4) (http://tree.bio.ed.ac.uk/software/figtree/).

For the analysis of the presence and absence of DNAJC9 homologs in fungal species, the number of proteins containing the PTHR44144 domain in each fungal species included in the Ensembl Fungi database (http://fungi.ensembl.org/) was retrieved using BioMart in Ensembl Fungi. A time-calibrated phylogenetic tree of representative fungal species was obtained from the TimeTree resource (http://www.timetree.org/).

## Results

### Loss of Djc9 renders Asf1 non-essential

In *S. pombe*, the gene encoding Asf1 (also known as Cia1) is essential for cell growth (Supplementary Fig. S1A) [12]. However, loss of Asf1 does not severely impact cell growth in the budding yeast *S. cerevisiae* [11]. It has been shown that approximately 65% of genes essential in *S. pombe* but not in *S. cerevisiae* can have their essentiality in *S. pombe* bypassed by suppressor mutations [60,61]. Thus, we hypothesized that the essentiality of *S. pombe asf1* may be bypassable.

**Figure 1.**
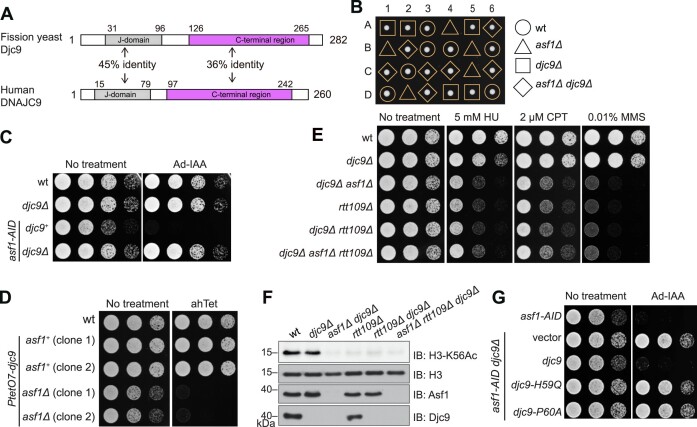
Loss of the DNAJC9 ortholog Djc9 bypasses the essentiality of the *asf1* gene in *Schizosaccharomyces pombe*. (**A**) Domain architectures of fission yeast Djc9 and human DNAJC9. We note that the annotation of the start codon of the *djc9* gene at PomBase has recently been revised, and the currently annotated start codon of *djc9* corresponds to the second methionine (M28) of the amino acid sequence of Djc9 used in this study. (**B**) Tetrad dissection showing that *djc9Δ* can suppress the lethality of *asf1Δ*. Six tetrads from one cross are shown, and four progeny from each of the tetrads are labeled as A, B, C, and D. (**C**) The inviability caused by AID-mediated Asf1 depletion was suppressed by *djc9Δ*. Five-fold serial dilutions of cells with indicated genotypes were spotted on YES plates with or without Ad-IAA. (**D**) Placing *djc9* under the control of an ahTet-inducible promoter rendered *asf1Δ* non-lethal in medium without ahTet. (**E**) *asf1Δ djc9Δ* cells exhibited sensitivities to genotoxic stresses. Five-fold serial dilutions of cells with indicated genotypes were spotted on a YES plate and YES plates containing HU, CPT, or MMS. (**F**) *asf1Δ djc9Δ* cells were defective in H3-K56 acetylation. Equal amounts of cells with the indicated genotypes were collected and whole cell extracts were examined by immunoblotting using antibodies against H3-K56Ac, H3, Asf1, or Djc9. (**G**) The HPD motif in the J-domain is required for the toxicity of Djc9 to Asf1-depleted cells. Wild-type and mutant Djc9 under the control of the *djc9* promoter were separately introduced into an *asf1-AID djc9Δ* strain. The growth phenotype in the absence or presence of Ad-IAA was analyzed.

To identify potential suppressors that can convert *asf1* from an essential gene to a non-essential one, we used a *piggyBac* transposon insertional mutagenesis-based strategy to perform bypass-of-essentiality (BOE) suppressor screening [61]. We pooled mutant clones that can tolerate the loss of *asf1* and sequenced the transposon insertion junctions. Multiple independent insertions within the coding sequence of a yet uncharacterized gene *SPAC1071.09c* were identified (Supplementary Fig. S1B). *SPAC1071.09c* encodes the ortholog of human DNAJC9, and therefore we named it *djc9*. The homologs of DNAJC9 and Djc9 are present across eukaryotes with the exception of some budding yeast species including *S. cerevisiae* (Supplementary Fig. S1C and D). Djc9 and DNAJC9 both contain an N-terminal J-domain, which is a binding domain for Hsp70, and share substantial sequence similarity in their C-terminal regions (Fig. 1A).

Consistent with growth phenotype data from a genome-wide gene deletion analysis [62], we found that *djc9* deletion did not cause an obvious growth defect (Supplementary Fig. S1E). To verify whether disrupting the *djc9* gene can suppress the growth defect caused by *asf1* functional loss, we introduced *djc9* deletion into two different temperature-sensitive (ts) *asf1* mutants (*asf1-30* and *asf1-33*) [63, 64], *djc9* deletion completely suppressed the growth defect of these *asf1* ts mutants at the restrictive temperature (Supplementary Fig. S1E). Furthermore, we found that *asf1* can be readily deleted in a *djc9* deletion background. We crossed an *asf1Δ djc9Δ* double deletion strain with a wild-type strain and performed tetrad dissection analysis (Fig. 1B). Among the cross progeny, the ones harboring only *asf1* deletion were inviable whereas the ones harboring both *asf1* deletion and *djc9* deletion were viable. Remarkably, the double deletion clones formed colonies of the same sizes as the wild-type clones, indicating that loss of Djc9 not only renders *asf1Δ* cells viable, but also renders the vegetative growth of *asf1Δ* cells completely normal.

In addition, we fused an auxin-inducible degron (AID) to the C-terminus of Asf1 at the endogenous locus so that Asf1 can be rapidly depleted upon the addition of 5-adamantyl-IAA (Ad-IAA) to the medium [39]. Consistent with the above observations, *asf1-AID* cells failed to grow on a medium containing Ad-IAA and deletion of *djc9* completely suppressed this growth defect (Fig. 1C). Moreover, we constructed a *PtetO7-djc9* strain by replacing the endogenous *djc9* promoter with a *PtetO7* promoter that can be induced by anhydrotetracycline (ahTet) (Supplementary Fig. S1F) [42]. *asf1* deletion in this strain was not lethal in the absence of ahTet, but did cause a mild growth defect, likely due to a leaky expression of Djc9 (Fig. 1D, Supplementary Fig. S1F). The presence of ahTet completely blocked the growth of the *PtetO7-djc9 asf1Δ* strain (Fig. 1D). Together, these results indicate that the presence or absence of Djc9 determines whether *asf1* is essential or not in fission yeast. In other words, Djc9 is toxic and causes lethality in the absence of Asf1 but is benign in the presence of Asf1.

Due to the lethality of *asf1Δ* in *S. pombe*, whether Asf1 contributes to histone H3K56 acetylation in *S. pombe* has been unknown. Even though the *asf1*Δ *djc9Δ* double deletion mutant exhibited no obvious growth phenotype in standard media, it showed strong sensitivity to the DNA replication inhibitor HU and the DNA damaging drugs methylmethanesulfonate (MMS) and camptothecin (CPT) (Fig. 1E). These sensitivity phenotypes are similar to those of the mutant lacking Rtt109, the histone acetyltransferase that catalyzes H3K56 acetylation (Fig. 1E). Furthermore, the *asf1*Δ *djc9Δ rtt109*Δ triple deletion mutant did not show stronger sensitivity than *asf1*Δ *djc9Δ*. Immunoblotting analysis of whole cell extracts showed that H3K56 acetylation was not affected in *djc9Δ* but was abolished in both *rtt109*Δ and *asf1*Δ *djc9Δ* (Fig. 1F), indicating that Asf1 is essential for H3K56 acetylation in fission yeast.

Because Djc9 harbors a J-domain, we tested whether its function requires the Hsp70-binding ability of the J-domain. We mutated the His-Pro-Asp (HPD) motif within the J-domain, which is a motif essential for Hsp70 binding [28]. As expected, Djc9 can be co-immunoprecipitated with the Hsp70 protein Ssa1 and mutating either of two HPD residues (H59Q and P60A) abolished the ability of Djc9 to interact with Ssa1 (Supplementary Fig. S1G). *In vivo*, these HPD mutations also prevented Djc9 from hindering the growth of *asf1-AID djc9Δ* cells in the presence of Ad-IAA (Fig. 1G). Based on these results, we concluded that Djc9 depends on its functional cooperation with Hsp70 to exert a toxic effect on Asf1-deficient cells.

Given that Djc9 and its human ortholog DNAJC9 possess highly conserved J-domains and significantly conserved C-terminal regions, we investigated whether human DNAJC9 could complement the role of Djc9 in inhibiting the growth of Asf1-depleted cells. DNAJC9 was expressed in *asf1-AID djc9Δ* cells using the ahTet-inducible *PenotetSW2* promoter (Supplementary Fig. S1H). Induction of DNAJC9 expression with ahTet strongly inhibited cell growth in the presence of Ad-IAA (Supplementary Fig. S1I), indicating that human DNAJC9 shares with Djc9 the ability to confer toxicity to Asf1-depleted fission yeast cells.

### Structural basis of the interactions between Djc9 and histones H3–H4

**Figure 2.**
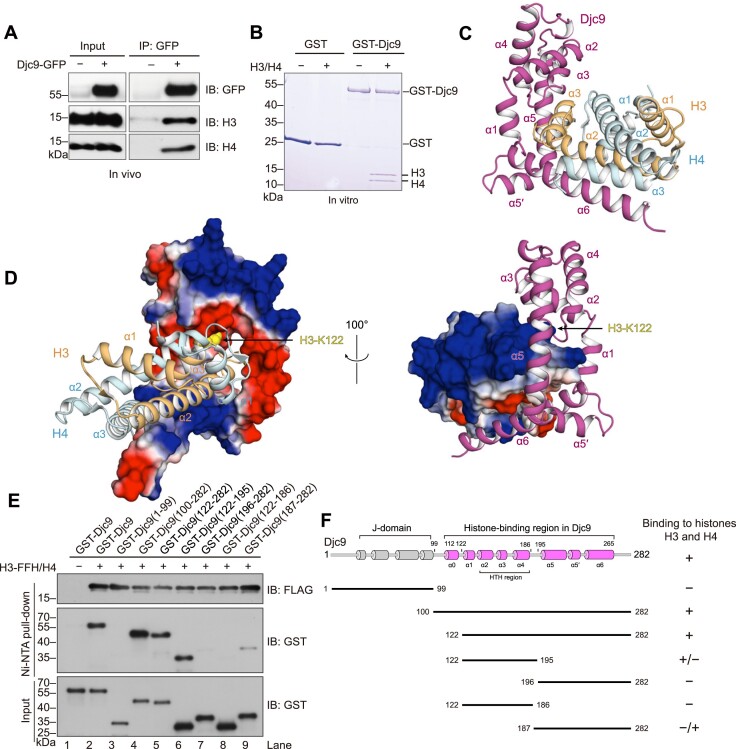
Djc9 interacts with the H3–H4 dimer through an interface much larger than the known histone-binding interface of human DNAJC9. (**A**) Co-IP of histones H3 and H4 with Djc9. Djc9 tagged with GFP at the C-terminus was expressed from the *Pnmt1* promoter and immunoprecipitated by GBP beads. Co-immunoprecipitated histones H3 and H4 were detected by immunoblotting using histone antibodies. (**B**) Djc9 directly interacts with histones H3 and H4. An *in vitro* GST pull-down assay was performed using recombinant H3, H4, and GST-tagged Djc9 purified from *Escherichia coli*. GST was used as a control. (**C**) The AlphaFold-Multimer-predicted structure of the α6-bound conformation of the Djc9–H3–H4 complex. For clarity, the confidently predicted (pLDDT > 70) histone-binding C-terminal region of Djc9 (residues 125–268) and confidently predicted regions of histones (residues 60–135 of H3 and residues 24–100 of H4) are shown. (**D**) Electrostatic surface views of Djc9 (left) and histones H3–H4 (right) in the predicted structure of the α6-bound conformation of the Djc9–H3–H4 complex. Surfaces of proteins are colored according to residue charge (blue for positive, red for negative, and white for neutral). In the cartoon view of histones (left), the H3-K122 residue is shown in a sphere representation. (**E**) Analyzing the histone-binding ability of truncated Djc9 fragments using *in vitro* His-tag pull-down. GST-tagged Djc9 was co-expressed with FLAG_2_-His_6_ (FFH)-tagged H3 and untagged H4 in *E. coli*. GST-Djc9 expressed alone served as a negative control. Ni-NTA resin was used to pull down H3-FFH. Both the pull-down and input fractions were analyzed by immunoblotting. (**F**) Diagram depicting the results of the truncation analysis shown in E. The secondary structure composition of Djc9, as predicted by AlphaFold, is shown at the top. The HTH region, consisting of helices α2-α4, is indicated by a bracket.

Like its human ortholog DNAJC9, Djc9 interacts with histones H3 and H4 both *in vivo* and *in vitro* (Fig. 2A and B). To understand how Djc9 interacts with H3 and H4, we employed AlphaFold-Multimer for structural prediction [49]. When using full-length Djc9, H3, and H4 as input, AlphaFold-Multimer successfully predicted the structure of a Djc9–H3–H4 complex, in which only the C-terminal region of Djc9 downstream of the J domain interacts with the H3–H4 dimer (Supplementary Fig. S2A and B). In this structure, the C-terminal region of Djc9 consists of seven α-helices, denoted as α0 to α6 here. α0 to α5 but not α6 are confidently predicted, and α6 does not make contact with histones or other parts of Djc9. DALI search did not identify any overall structural similarity between the C-terminal region of Djc9 and known structures but revealed that helices α2-α4 adopt a tri-helical helix-turn-helix (HTH) conformation [65].

A previous study identified in human DNAJC9 a minimal histone-binding region, called histone-binding domain (HBD), and solved a crystal structure of the HBD–H3.3–H4–MCM2 complex [32]. DNAJC9 HBD is composed of two α-helices, referred to as αA and αB. These two helices correspond to α5 and α6 of Djc9. When we compared the two structures, α5 of Djc9 can be superimposed onto αA of DNAJC9 (Supplementary Fig. S2C). In contrast, α6 of Djc9 does not occupy the same position as αB of DNAJC9. Instead, α0 of Djc9 occupies a position similar to that of αB of DNAJC9, suggesting that α0 and α6 of Djc9 may compete for binding with H3–H4 (Supplementary Fig. S2C). To test this idea, we used an N-terminally truncated Djc9 sequence, Djc9(122–282), which lacks the sequence of α0, as input for AlphaFold-Multimer prediction. In the resulting structure, the α6 helix has a high confidence score, is in contact with H3–H4, and occupies the same position as αB of DNAJC9 (Supplementary Fig. S2D–F). We call the two different histone-binding conformations of Djc9 the α0-bound conformation and the α6-bound conformation, respectively. The α6-bound conformation has an extra α-helix between α5 and α6, and we call it α5′.

We also applied AlphaFold-Multimer on human DNAJC9, H3.3, and H4. Similar to the situation with the fission yeast proteins, two different histone-binding conformations of DNAJC9 were predicted depending on whether the full-length or an N-terminally truncated sequence of DNAJC9 was used as input (Supplementary Fig. S2G–L). Overall, each conformation is highly similar to the corresponding conformation of Djc9.

In both the α0-bound conformation and the α6-bound conformation, the histone-binding regions of Djc9 and DNAJC9 are much larger than the HBD in DNAJC9 (Supplementary Fig. S2C, F, I, and L). In Fig. 2C and D, we illustrate in detail the α6-bound conformation of Djc9, as this conformation is important for the *in vivo* function of Djc9 (see below). In this conformation, the interaction interface between Djc9 and the H3–H4 dimer has a buried surface area of 2204 Å^2^. The interface can be divided into two sub-interfaces. Sub-interface I involves helices α1-α5′ of Djc9, which form a doughnut shape. One end of the ellipsoid-shaped H3–H4 dimer points toward the doughnut hole and makes extensive contacts with the α6-facing surface of the doughnut. Djc9 residues participating in this sub-interface mainly belong to α1, α3, α5, and the loop between α3 and α4. Histone residues participating in this sub-interface mainly belong to α3 and the loop preceding α3 in H3. Sub-interface II involves Djc9 α6, H3 α2, and H4 α3, together forming a three-helix bundle conformation (Fig. 2C). Both sub-interfaces show charge complementarity. Djc9 residues on sub-interface I are enriched with acidic ones, which interact with a positively charged H3 surface, where the Lys122 residue of H3 contacts the surface lining the doughnut hole of Djc9 (Fig. 2D). In contrast, for sub-interface II, the histone-interacting surface of Djc9 α6 is enriched with positively charged residues, which contact negatively charged residues on H3 α2 (Fig. 2D).

To verify the structures predicted by AlphaFold-Multimer, we performed *in vitro* pull-down experiments using truncated Djc9 (Fig. 2E and F). Consistent with the predicted structures, the C-terminal region of Djc9 (residues 100–282), but not the N-terminal J-domain (residues 1–99), interacted with histones H3–H4. Djc9(122–282), which lacks α0, can still efficiently bind H3–H4, supporting the α6-bound conformation of the Djc9–H3–H4 complex. Two shorter fragments, Djc9(122–195) and Djc9(187–282), the latter of which corresponds to the HBD of human DNAJC9, exhibited weakened but still detectable H3–H4 binding, consistent with the fact that both fragments contain residues involved in histone binding in the predicted structures.

We then used point mutations at the histone-binding surface of Djc9 to further verify the predicted complex structures. Mutating two hydrophobic residues on the H3-α3-binding surface of Djc9 (sub-interface I), Leu129 and Phe132, to arginines notably weakened the ability of Djc9(100–282) to bind H3–H4 (Supplementary Fig. S3A). Mutating either of the two pairs of α6 residues (L242A/I246A or L261R/Y265R) on sub-interface II diminished the histone binding ability of Djc9(187–282) (Supplementary Fig. S3B), supporting a role of α6 in histone binding.

To assess the physiological roles of the two histone-binding conformations, we examined whether mutating histone-binding residues in predicted interfaces affects the toxicity of Djc9 in the *asf1-AID djc9Δ* background (Supplementary Fig. S3C and D). Mutating the H3-α3-binding surface of Djc9 abolished the ability of Djc9 to curtail the growth of *asf1-AID djc9Δ* cells (Supplementary Fig. S3C). Deletion of the Djc9 α0 (removing residues 110–120) only marginally affected the toxicity of Djc9, whereas the α6 mutations L261R or l241A/I246A severely weakened the toxicity of Djc9, suggesting that α6-mediated histone interaction plays a more important role than α0-mediated histone interaction in inhibiting cell growth in the absence of Asf1 (Supplementary Fig. S3D). Interestingly, deletion of the Djc9 linker region (100–110) between the J-domain and the histone-binding regions weakened the toxicity of Djc9 more than the α0 deletion, and deleting both the linker and α0 strongly diminished the toxicity, suggesting that a proper spacing between the J-domain and the C-terminal histone-binding region is important. Together, these results demonstrate that the toxicity of Djc9 to Asf1-deficient cells depends on its histone-binding ability.

### Asf1 competes with Djc9 for binding to histones H3–H4

**Figure 3.**
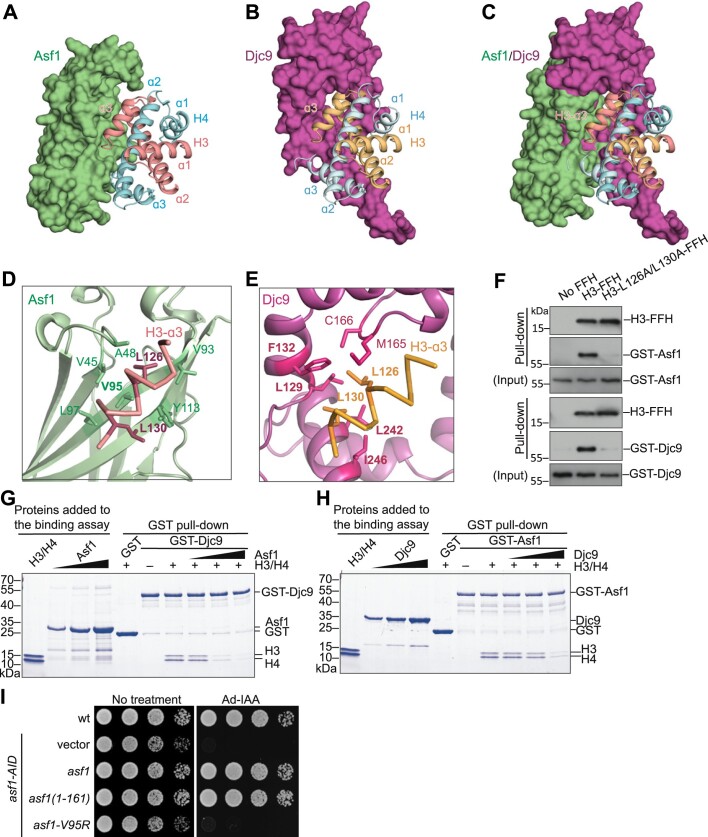
Djc9 and Asf1 interact with the H3–H4 dimer in a mutually exclusive manner. (A-C) Comparison of the AlphaFold-Multimer-predicted structures of the Asf1–H3–H4 complex and the Djc9–H3–H4 complex: (**A**) the structure of the Asf1–H3–H4 complex with Asf1 shown in a surface representation and H3–H4 shown in a cartoon representation; (**B**) the structure of the Djc9–H3–H4 complex with Djc9 shown in a surface representation and H3–H4 shown in a cartoon representation; (**C**) two complex structures superimposed based on H3. (**D**) Close-up view of the interactions between two interface residues on the α3-helix of histone H3 (L126 and L130, represented by sticks) and the residues of Asf1. Asf1 residues that are within 5 Å of H3-L126 or H3-L130 and with their side chains facing H3-L126 or H3-L130 are shown in stick representation. Asf1-V95, whose mutation was analyzed in the experiment shown in panel G, is highlighted in bold font. (**E**) Close-up view of the interactions between L126 and L130 of histone H3-α3 and the residues of Djc9. Djc9 residues that are within 5 Å of H3-L126 or H3-L130 and with their side chains facing H3-L126 or H3-L130 are shown in stick representation. The four Djc9 residues whose mutations were analyzed in the experiments shown in Supplementary Fig. S3 are highlighted in bold font. (**F**) Mutating H3-L126 and H3-L130 to alanines disrupted the interaction between H3–H4 and Asf1, and the interaction between H3–H4 and Djc9. Ni-NTA pull-downs were performed as in Fig. 2F. (G and H**)***In vitro* binding assay showing competition between Djc9 and Asf1 for H3–H4 binding. Glutathione agarose-bound GST-Djc9 (**G**) or GST-Asf1 (**H**) was mixed with purified H3–H4 in the absence or presence of increasing amounts of Asf1 (**G**) or Djc9 (**H**). After incubation, beads-bound proteins were visualized by Coomassie Brilliant Blue (CBB) staining of SDS-PAGE gel. (**I**) The histone-binding residue V95 of Asf1 is required for its essential function. Wild-type Asf1, the Asf1 core domain (residues 1–161), or the V95R mutant of Asf1 under the native promoter was expressed in *asf1-AID* cells. Growth in the absence or presence of Ad-IAA was analyzed.

Both Asf1 and Djc9 are binders of the histone H3–H4 dimer. It has previously been shown that helix αB of DNAJC9 HBD (corresponding to α6 of Djc9) forms a steric clash with Asf1 when comparing the structures of the DNAJC9–H3–H4 complex and the ScAsf1–H3–H4 complex [66]. Whether DNAJC9 and Asf1 bind H3–H4 in a mutually exclusive manner has not been experimentally examined. Comparing the AlphaFold-predicted structures of the Djc9–H3–H4 complex (α6-bound conformation) and the SpAsf1–H3–H4 complex, we found that the region of Djc9 that binds H3-α3 presents a major steric clash with Asf1 (Fig. 3A–C). In the predicted structures, two hydrophobic residues in H3-α3, Leu126 and Leu130, bind to a hydrophobic groove of Asf1 in the Asf1–H3–H4 complex (Fig. 3D) [5], and pack against a hydrophobic surface of Djc9 in the Djc9–H3–H4 complex (Fig. 3E). Consistent with the structures, pull-down assays showed that mutating both Leu126 and Leu130 of H3-α3 to alanine substantially weakened both the interaction between H3–H4 and Asf1 and the interaction between H3–H4 and Djc9 (Fig. 3F).

We directly tested whether Asf1 and Djc9 bind to H3–H4 in a competitive manner using purified proteins, and found that, the addition of Asf1 inhibited the binding of histones H3–H4 by Djc9 in a dose-dependent manner (Fig. 3G), and vice versa (Fig. 3H). Thus, as predicted by the structures, Asf1 and Djc9 compete for H3–H4 binding. Because in *S. pombe* Asf1 is only essential when Djc9 is present, we proposed that the essential function of Asf1 is to competitively inhibit H3–H4 binding by Djc9. Consistent with this idea, the mutation of a residue in the H3-α3-binding groove of Asf1 (Fig. 3D), *asf1-V95R*, which is known to disrupt the histone-binding ability of Asf1 [4, 67], abolished the ability of Asf1 to rescue *asf1-AID* (Fig. 3I). These results suggest that the essential function of Asf1 in *S. pombe* is to compete with Djc9 for histone binding.

### A histone H3 point mutation bypasses the essentiality of Asf1 through abolishing the histone–Djc9 interaction

If the toxic effect exerted by Djc9 on Asf1-deficient cells requires the Djc9–histone interaction, we hypothesized that the growth defect of Asf1-deficient cells should be suppressible by histone mutations that abolish the Djc9–histone interaction but still maintain chromatin functions. To identify such histone mutations, we introduced random mutations into an H3–H4-expressing plasmid by error-prone RCA [51], transformed the mutagenized plasmid DNA into an *asf1-30* strain, and screened for clones that can grow at a restrictive temperature. The only mutation present in multiple independent clones obtained from this screen is a missense mutation in H3, converting lysine 122 to glutamate (H3-K122E).

**Figure 4.**
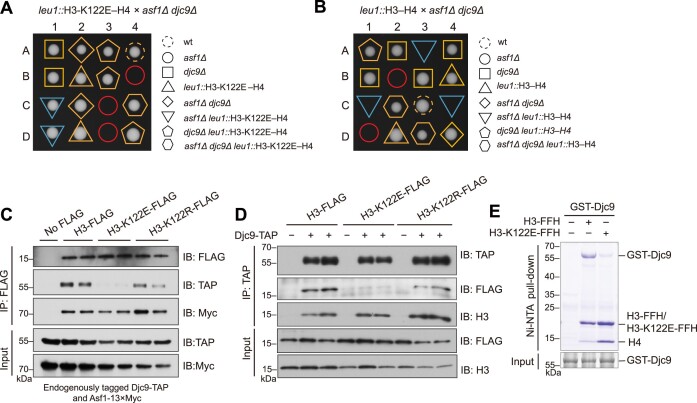
The lethality of *asf1Δ* is suppressed by the H3-K122E mutation that disrupts the histone-Djc9 interaction. (A and B) Tetrad dissection showing that the expression of H3-K122E (**A**) but not wild-type H3 (**B**) from an integrating plasmid suppresses the lethality of *asf1Δ*. H3 and H4 were expressed from the same plasmid under the control of their native promoters. (C and D) Reciprocal IP showing that H3-K122E is defective in Djc9 binding. C-terminally FLAG-tagged wild-type H3, H3-K122E, or H3-K122R were expressed together with H4 under their native promoters in cells expressing endogenously TAP (Tandem Affinity Purification) -tagged Djc9 and endogenously 13 × Myc-tagged Asf1. The IP was performed using anti-FLAG M2 beads (**C**) or IgG beads (**D**). Immunoprecipitated proteins were analyzed by immunoblotting. (**E**) *In vitro* Ni-NTA pull-down assay showing that H3-K122E is defective in Djc9 binding. GST-Djc9, H3-FFH, and H4 were co-expressed in *E. coli*. Both the pull-down and input were analyzed by SDS-PAGE and CBB staining.

To test whether the H3-K122E mutation is able to bypass the essentiality of Asf1, we crossed a wild-type strain carrying an integrating plasmid expressing H3-K122E with an *asf1Δ djc9Δ* double deletion mutant strain, and examined the growth phenotype of the cross progeny by tetrad analysis. The *asf1Δ* single deletion mutant containing the H3-K122E plasmid grew as well as the wild type and *asf1Δ djc9Δ* double deletion mutant (Fig. 4A). The control plasmid expressing wild-type H3 failed to rescue the lethality of *asf1Δ* (Fig. 4B). Thus, the presence of H3-K122E renders Asf1 dispensable, presumably by alleviating the toxicity of Djc9 when Asf1 is absent.

Lysine 122 of H3 has been reported to undergo post-translational modifications. In *S. cerevisiae*, the ubiquitination of this residue promotes the assembly of newly synthesized histones onto replicated DNA [68]. Acetylation of this residue in human cell lines and *S. pombe* was shown to stimulate transcription through destabilizing histone-DNA interactions [69]. To determine whether the ability of H3-K122E to rescue *asf1Δ* is due to the loss of post-translational modifications, we mutated Lys122 to arginine, which cannot be modified by ubiquitination and acetylation. Unlike H3-K122E, H3-K122R could not rescue the lethality of *asf1Δ* (Supplementary Fig. S4A). In addition, only H3-K122E but not H3-K122R could suppress the growth defect caused by Djc9 induction in *PtetO7-djc9 asf1Δ* cells (Supplementary Fig. S4B). Therefore, ectopic expression of an H3 mutant containing a charge-reversal mutation at the Lys122 residue protects *asf1Δ* cells from the toxicity of Djc9, whereas ectopic expression of the H3-K122R mutant that can no longer be ubiquitinated or acetylated at this residue is insufficient to confer protection.

Based on the rationale for the screen that identified the H3-K122E mutation, this mutation should abolish or weaken the histone–Djc9 interaction but not severely disrupt the other functions of H3. Indeed, H3-K122E could support growth as the only form of H3 in the cells, albeit the growth was slower than the control strain expressing wild-type H3 (Supplementary Fig. S4C and D), indicating that H3-K122E retains basic histone functions.

To determine the relationship between the H3-K122E mutation and the histone–Djc9 interaction, we first inspected the AlphaFold-predicted structure (α6-bound conformation) of the Djc9–H3–H4 complex. In this structure, the side chain of H3-K122 docks into an acidic concave surface formed by the turn of the HTH region of Djc9 (Fig. 2D, Supplementary Fig. S4E), indicating that mutating H3-K122 to an acidic residue is likely to generate charge repulsion and weaken the Djc9–H3 interaction. Consistent with this idea, using IP analysis, we found that the H3-K122E but not H3-K122R mutation dramatically diminished the *in vivo* interaction between H3–H4 and Djc9 (Fig. 4C and D). In contrast, neither H3-K122E nor H3-K122R affected the interaction between H3–H4 and Asf1 (Fig. 4C). Moreover, *in vitro* Ni-NTA pulldown assay using bacterially expressed proteins confirmed that the H3-K122E mutation strongly weakened the binding between H3–H4 and Djc9 (Fig. 4E). Thus, these results demonstrate that H3-K122E rescued the lethality of *asf1Δ* by disrupting the interaction between H3–H4 and Djc9.

### Djc9 causes downregulation of histones H3 and H4 in the absence of Asf1

**Figure 5.**
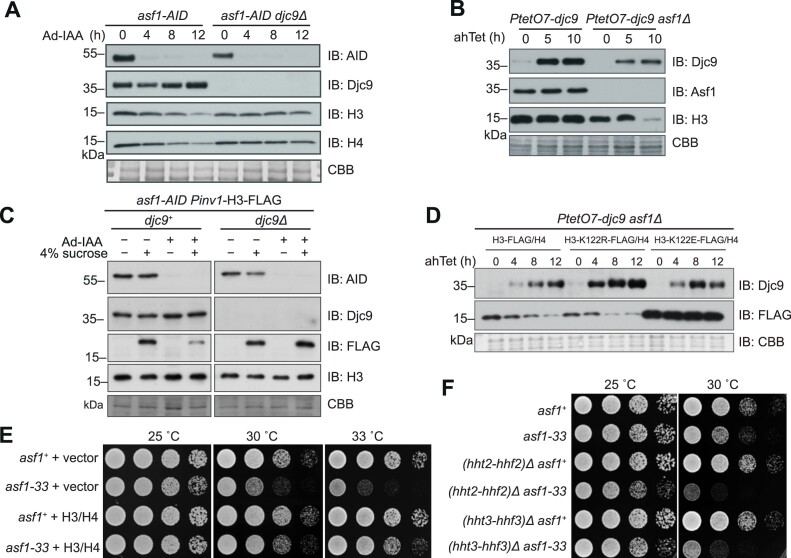
Djc9 causes H3–H4 degradation upon Asf1 inactivation. (**A**) Immunoblotting showing that the levels of histones H3 and H4 were reduced upon Asf1 depletion in a Djc9-dependent manner. The levels of endogenous H3 and H4 were examined by immunoblotting analysis of whole cell extracts of *asf1-AID* and *asf1-AID djc9Δ* cells before and at the indicated time points after Ad-IAA treatment. (**B**) Immunoblotting showing that the induction of Djc9 in *PtetO7-djc9 asf1Δ* cells resulted in a reduction of endogenous H3. Cells were harvested before and at the indicated time points after Djc9 induction and whole cell extracts were subjected to immunoblotting analysis. (**C**) Immunoblotting showing that in Asf1-depleted cells, Djc9 restricts the accumulation of newly synthesized H3. FLAG-tagged H3 under the *Pinv1* promoter was induced by shifting cells from 8% glucose medium to 4% sucrose medium. Asf1-depleted samples were pre-treated with Ad-IAA for 1 h before the shift and then shifted to Ad-IAA-containing sucrose medium. Cells were collected 1 h after the shift and whole cell extracts were analyzed using immunoblotting with antibodies against AID, FLAG, Djc9, and H3. (**D**) Immunoblotting showing that the levels of ectopically expressed wild-type H3 and H3-K122R, but not H3-K122E, were reduced upon the induction of Djc9 in *PtetO7-djc9 asf1Δ* cells. Cells were harvested before and at different time points after Djc9 induction and whole cell extracts were subjected to immunoblotting analysis. (**E**) Increasing the dosage of H3 and H4 genes mitigated the growth defect of a temperature-sensitive *asf1* mutant. One extra copy of the H3–H4 gene pair was introduced into the wild-type and *asf1-33* mutant, and the growth phenotype at different temperatures was analyzed using a spot assay. (**F**) Reducing the dosage of H3 and H4 genes exacerbated the growth defect of a temperature-sensitive asf1 mutant. Either the *hhf2-hht2* gene pair or the *hhf3-hht3* gene pair was deleted in the *asf1-33* mutant background, and the growth phenotype at different temperatures was analyzed using a spot assay.

Given that the toxic effect exerted by Djc9 on Asf1-deficient cells depends on its interaction with histones H3 and H4, we investigated whether the toxicity caused by Djc9 is associated with changes in these histones. We first examined the levels of endogenous histones H3 and H4 in *asf1-AID* cells. Immunoblotting revealed that, upon Asf1 depletion, the levels of histones H3 and H4 gradually decreased (Fig. 5A). In contrast, no reduction in histones H3 and H4 was observed when Asf1 was depleted in the *djc9Δ* background. Moreover, in *asf1Δ PtetO7-djc9* cells, ahTet-induced expression of Djc9 led to a decrease in endogenous H3 levels, while no reduction in H3 levels occurred when Djc9 was induced in *PtetO7-djc9* cells (Fig. 5B). To determine whether Djc9 specifically promotes the reduction of histones H3 and H4, we examined the protein levels of all core histones in the *asf1Δ PtetO7-djc9* strain. We found that ahTet-induced expression of Djc9 caused a substantial reduction in the protein levels of H3 and H4, whereas the levels of H2A and H2B remained largely unchanged (Supplementary Fig. S5A). Therefore, the toxicity of Djc9 in the absence of Asf1 is associated with a specific reduction in the protein levels of histones H3 and H4.

Given the important role of Asf1 in chaperoning newly synthesized histones H3–H4, we wondered whether Djc9 mainly affects newly synthesized histones H3–H4 when Asf1 is depleted. To directly examine the behavior of newly synthesized histones, we ectopically expressed C-terminally FLAG-tagged H3 under the control of the *inv1* promoter (*Pinv1*), which can be rapidly induced within 1 h after switching from a glucose-containing medium to a sucrose-containing medium (Supplementary Fig. S5B) [70–72]. In *asf1-AID* cells, when Asf1 was depleted by the addition of Ad-IAA before the induction of *Pinv1*-controlled H3-FLAG, the induced protein level of H3-FLAG was substantially lower than that in cells without Asf1 depletion (Fig. 5C), although the induced transcript levels were similar (Supplementary Fig. S5B). In contrast, in *djc9Δ asf1-AID* cells, the protein level of H3-FLAG was induced to a similar level with or without Asf1 depletion (Fig. 5C). Unlike H3-FLAG expressed from the *Pinv1* promoter, the level of endogenous H3 was not affected by acute Asf1 depletion (Fig. 5C). These results support the model that in the absence of Asf1, Djc9 downregulates newly synthesized histones at the protein level.

Djc9-mediated downregulation of histones in Asf1-deficient cells requires the Djc9–histone interaction, as we found that in *asf1Δ PtetO7-djc9* cells, ahTet-induced expression of Djc9 resulted in the reduction of the level of ectopically expressed H3-K122R but not H3-K122E (Fig. 5D). Moreover, in *asf1Δ* cells rescued by an integrating plasmid expressing H3-K122E-FLAG, the level of endogenous H3 was markedly lower than that in *asf1Δ djc9Δ* cells containing the same plasmid, whereas the level of H3-K122E-FLAG was similar in both strains (Supplementary Fig. S5C). Additionally, Djc9-mediated histone downregulation in Asf1-deficient cells also requires the HPD motif in the J-domain of Djc9, as histone level reduction did not occur in *asf1Δ PtetO7-djc9-H59Q* cells upon ahTet-induced expression of Djc9-H59Q (Supplementary Fig. S5D).

If the growth defect of Asf1-deficient cells is caused by excessive histone downregulation, we predicted that increasing histone gene dosage may improve the growth of Asf1-deficient cells. Indeed, introducing one extra copy of the H3–H4 gene pair mitigated the growth defect of two different *asf1* ts mutants at the restrictive temperature (Fig. 5E, Supplementary Fig. S5E). Conversely, removing one of the three pairs of endogenous H3–H4 genes exacerbated the growth phenotype of *asf1* ts mutants (Fig. 5F, Supplementary Fig. S5F). Taken together, our results suggest that Djc9-mediated histone downregulation underlies the role of Djc9 in inhibiting cell growth in the absence of Asf1.

### Djc9 confers resistance to HU by promoting histone H3 and H4 degradation

**Figure 6.**
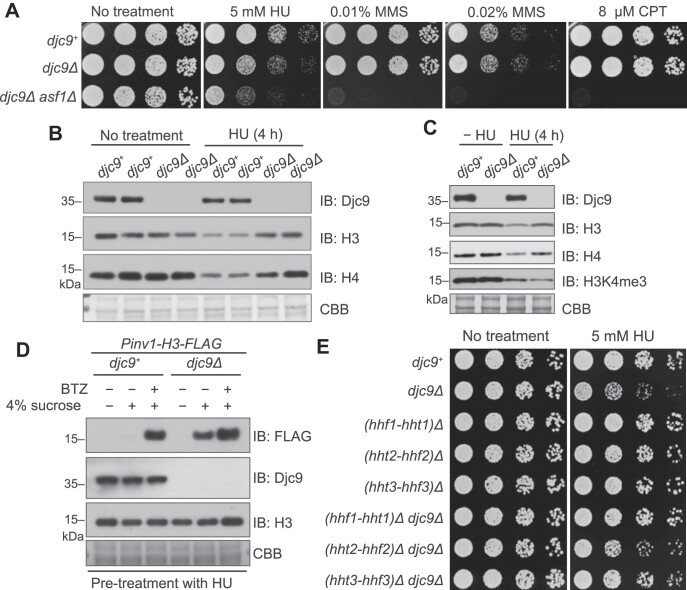
Djc9 confers HU resistance by promoting H3–H4 degradation. (**A**) *djc9Δ* cells exhibited sensitivity to the DNA replication inhibitor HU. Five-fold dilutions of cells were spotted on YES plates and YES plates containing HU, CPT, or MMS. Plates were scanned after 2 days of incubation at 30°C. (**B**) The levels of histones H3 and H4 were reduced upon HU treatment in a Djc9-dependent manner. The levels of endogenous H3 and H4 were examined by immunoblotting analysis of whole cell extracts of wild-type and *djc9Δ* cells treated or not treated with 15 mM of HU. Two clones were analyzed for each genotype. (**C**) Immunoblotting with H3K4me3-specific antibodies showing that pre-existing histones with H3K4me3 modification decreased after HU treatment in both wild-type and *djc9Δ* cells. (**D**) Immunoblotting showing that newly synthesized FLAG-tagged H3 from the sucrose-inducible *Pinv1* promoter accumulated in HU-arrested *djc9Δ* cells but not in HU-arrested wild-type cells. This difference was abolished by the proteasome inhibitor BTZ. Cells grown in 8% glucose medium were pre-treated with HU for 4 h and then shifted to HU-containing 4% sucrose medium with or without BTZ. Cells were collected 1 h after the shift and whole cell extracts were analyzed using immunoblotting with antibodies against FLAG, Djc9, and H3. (**E**) Reducing the dosage of H3 and H4 genes suppressed the HU sensitivity of *djc9Δ* cells. The growth phenotype of strains with indicated genotypes was analyzed using a spot assay.

Even though *djc9Δ* does not obviously affect vegetative growth, we observed that *djc9Δ* cells exhibited a mild sensitivity to HU, which blocks DNA replication, suggesting that in the presence of Asf1, Djc9 plays a beneficial role when cells are challenged with HU (Figs 1E and 6A). Moreover, the expression of wild-type but not HPD motif-mutated Djc9 rescued the HU sensitivity of *djc9Δ* cells (Supplementary Fig. S6A), suggesting that Hsp70-binding is indispensable for Djc9 to confer HU resistance. To explore the mechanism underlying the HU sensitivity of *djc9Δ*, we compared the protein levels of H3 and H4 in wild-type cells versus *djc9Δ* cells. Under normal culturing conditions, the protein levels of H3 and H4 in wild-type cells were similar to those in *djc9Δ* cells (Fig. 6B). When wild-type cells were exposed to HU and arrested in S phase (Supplementary Fig. S6B), the protein levels of H3 and H4 were appreciably reduced compared to untreated cells (Fig. 6B). Interestingly, this reduction did not happen in *djc9Δ* cells treated with HU (Fig. 6B), despite a similarly efficient cell cycle arrest (Supplementary Fig. S6B). Thus, Djc9 is required for histone reduction in HU-arrested cells.

Immunoblotting analysis using an antibody specific to trimethylated histone H3 lysine 4 (H3K4me3), a modification present on preexisting histone H3 but not on newly synthesized histone H3 [73], showed that upon HU treatment, the level of H3K4me3 was reduced in both wild-type and *djc9Δ* cells (Fig. 6C). This result suggests that the difference in total H3 level between HU-treated *djc9Δ* cells and HU-treated wild-type cells is mainly due to the difference in the levels of newly synthesized H3. Because HU treatment prevents DNA replication but not the bulk of RNA and protein synthesis [74], and causes cell elongation (Supplementary Fig. S6C), the reduction in H3K4me3 level upon HU treatment likely reflects a reduction of the ratio of preexisting DNA-bound histones compared to total cellular proteins. If this idea is correct, we predicted that imposing a G1 phase arrest, which prevents both DNA replication and S-phase-specific histone expression but not the synthesis of most other proteins [75, 76], should also lead to a reduction in histone levels. Indeed, when we arrested cells in G1 using the temperature-sensitive *cdc10-129* mutation (Supplementary Fig. S6D), the protein levels of H3 and H4 decreased compared to unsynchronized cells (Supplementary Fig. S6E). Unlike HU treatment, G1 arrest-induced histone reduction still occurred in the absence of Djc9, supporting the idea that Djc9-dependent reduction of histone levels upon HU treatment happens to histones newly synthesized in S phase.

To directly examine the behavior of newly synthesized histones in HU-arrested cells, we examined H3-FLAG expressed from the sucrose-inducible *Pinv1* promoter. When cells were treated with HU for 4 h and then shifted to a medium containing sucrose and HU, H3-FLAG was robustly expressed in *djc9Δ* cells but was undetectable in wild-type cells (Fig. 6D). We hypothesized that the lack of detectable H3-FLAG in HU-arrested wild-type cells may be due to proteasome-mediated degradation. Indeed, when we added the proteasome inhibitor bortezomib (BTZ) to the sucrose- and HU-containing medium, H3-FLAG became detectable in HU-treated wild-type cells and the protein level of H3-FLAG is similar to that seen in HU-treated *djc9Δ* cells (Fig. 6D). To determine whether this role of Djc9 in promoting histone degradation in HU-treated cells requires its interaction with H3–H4, we examined the behavior of *Pinv1*-expressed H3-K122E-FLAG, which is defective in Djc9 binding. Unlike wild-type H3-FLAG, the protein level of H3-K122E-FLAG expressed during HU arrest was similar in wild-type and *djc9Δ* cells (Supplementary Fig. S6F). Taken together, these results indicate that Djc9 promotes the degradation of newly synthesized histones upon DNA replication blockage in a manner dependent on its interaction with H3–H4.

We hypothesized that the aberrant accumulation of newly synthesized histones H3 and H4 in *djc9Δ* cells may cause the HU sensitivity. To assess this possibility, we combined *djc9* deletion with the deletion of one of three pairs of H3–H4 genes. In concordance with a previous report, *S. pombe* strains lacking any one of three pairs of H3–H4 genes had no obvious growth phenotype (Fig. 6E) [77]. These strains also showed a normal level of HU sensitivity (Fig. 6E). Remarkably, deleting the gene pair *hht1-hhf1* or the gene pair *hht3-hhf3* completely suppressed the HU sensitivity phenotype of *djc9Δ*, whereas deleting the gene pair *hht2-hhf2* partially suppressed the HU sensitivity of *djc9Δ* (Fig. 6E). These results support the idea that Djc9 confers HU resistance though preventing the accumulation of newly synthesized histones.

### Djc9 confers resistance to dominant-negative forms of histone H3

**Figure 7.**
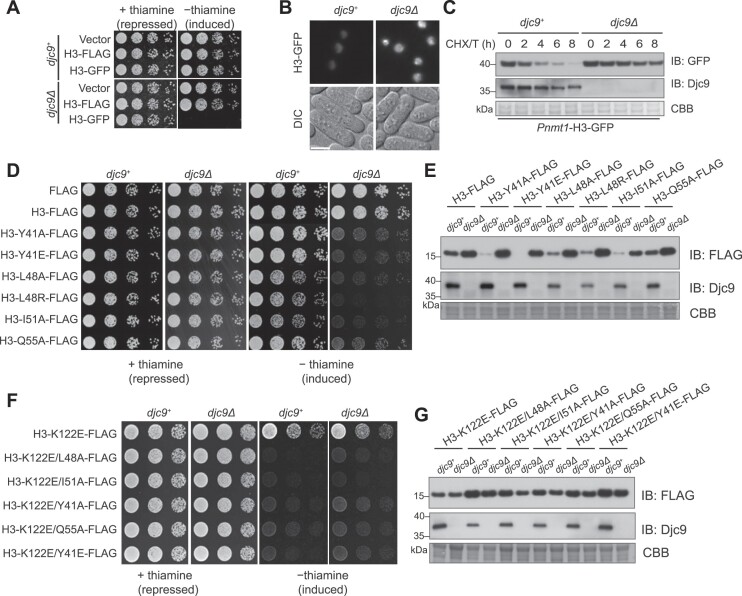
Djc9 protects cells from dominant-negative histone mutants. (**A**) The growth of *djc9Δ* cells, but not wild-type cells was severely inhibited by the ectopic expression of GFP-tagged H3 from a *Pnmt1* promoter. (**B**) Ectopically expressed GFP-tagged H3 from a *Pnmt1* promoter accumulated at a higher level in *djc9Δ* cells compared to wild-type cells. Cells were shifted to a thiamine-free medium for 20 h and then examined by fluorescence microscopy. DIC, differential interference contrast. Scale bar, 5 μm. (**C**) Immunoblotting showing faster turnover of H3-GFP in wild-type cells compared to *djc9Δ* cells. Cells were initially shifted to a thiamine-free medium for 16 h to induce the expression of H3-GFP from a *Pnmt1* promoter. Thiamine and 10 mg/mL of CHX were subsequently added to the culture. Cell aliquots were harvested at the indicated time points. Whole cell lysates were immunoblotted with the indicated antibodies. (**D**) The growth of *djc9Δ* cells, but not wild-type cells, was severely inhibited by the ectopic expression of several missense H3 mutants. The growth phenotype of wild-type cells and *djc9Δ* cells expressing *Pnmt1* promoter-driven wild-type or mutant versions of histone H3 was examined using a spot assay. (**E**) Djc9 prevents the accumulation of dominant-negative histone mutants in the cells. Wild-type or mutant forms of H3, tagged with FLAG at the C-terminus, were expressed from the *Pnmt1* promoter. Protein levels were examined by immunoblotting using the indicated antibodies. (**F**) The H3-K122E mutation, disrupting the Djc9-histone interaction, rendered dominant-negative histone mutants toxic to wild-type cells. (**G**) The H3-K122E mutation, disrupting the Djc9–histone interaction, allowed dominant-negative histone mutants to accumulate in wild-type cells to the same extent as in *djc9Δ* cells.

During our study we noticed that ectopic expression of C-terminally green fluorescent protein (GFP)-tagged histone H3 (H3-GFP) using a strong inducible promoter *Pnmt1* was toxic to *djc9Δ* but not wild-type cells (Fig. 7A). Live cell imaging showed that H3-GFP accumulated to a higher level in *djc9Δ* cells than in wild-type cells (Fig. 7B). We then monitored the turnover of H3-GFP by shifting cells from the induction condition (−thiamine) to a condition that prevents the expression of Djc9 (the presence of both thiamine and the protein synthesis inhibitor cycloheximide (CHX)). We observed that the turnover of H3-GFP was substantially faster in wild-type cells than in *djc9Δ* cells (Fig. 7C). These results suggest that Djc9 prevents the toxicity of H3-GFP by promoting its degradation.

We hypothesized that the ability of Djc9 to promote histone degradation may allow it to protect cells from the toxicity of not only H3-GFP but also other dominant-negative forms of histones. To identify such forms of histones, we selected missense H3 and H4 mutations that individually render H3 or H4 unable to support growth in *S. cerevisiae* [53, 78]. We ectopically expressed H3 or H4 harboring such mutations using the strong inducible promoter *Pnmt1* in *djc9Δ* cells. Five H3 mutants, Y41A, Y41E, L48A, I51A, and Q55A, potently inhibited the growth of *djc9Δ* cells but had no effect or only a mild effect on the growth of wild-type cells (Fig. 7D). An H3 mutation at position 48, L48R, has recently been identified as a likely disease-causing dominant mutation in a patient with neurodegenerative symptoms [79]. We found that H3-L48R also showed strong toxicity to *djc9Δ* when ectopically expressed (Fig. 7D).

If the strong toxicity of these dominant-negative H3 mutants to *djc9Δ* but not wild-type cells is due to Djc9 promoting their degradation, we predicted that these mutants should accumulate to higher levels in *djc9Δ* cells than in wild-type cells. Indeed, the protein levels of these H3 mutants expressed from the *Pnmt1* promoter were higher in *djc9Δ* cells than in wild-type cells (Fig. 7E). *Pnmt1* promoter-expressed wild-type H3, which is non-toxic, also accumulated to a higher level in *djc9Δ* cells. Interestingly, except for H3-Q55A, which behaved like wild-type H3, the other H3 mutants accumulated to substantially lower levels than wild-type H3 in the presence of Djc9, suggesting that they are degraded by Djc9 to a greater extent than wild-type H3.

To investigate whether this role of Djc9 in preventing the toxicity of dominant-negative H3 mutants requires its interaction with H3–H4, we examined the effect of the H3-K122E mutation, which disrupts the interaction. We observed that when the H3-K122E mutation was combined with the dominant-negative H3 mutations, the toxicity to wild-type cells became as strong as the toxicity to *djc9Δ* cells (Fig. 7F), and the protein levels no longer showed differences between wild-type cells and *djc9Δ* cells (Fig. 7G). Taken together, these results suggest that Djc9 protects the cells from the toxicity of dominant-negative forms of histones by promoting their degradation.

## Discussion

The roles of histone-binding proteins in facilitating chromatin assembly and disassembly have been extensively studied [80, 81]. Our study reveals that as a binding protein for the histone H3–H4 dimer, Djc9 acts with Hsp70 to promote the proteasomal degradation of excess histones. Its activity is restrained by the competitive binding of H3–H4 by Asf1, whose essential function in fission yeast is restricting histone degradation by Djc9. The loss of Djc9 causes sensitivities to conditions that lead to histone oversupply and renders Asf1 dispensable for cell proliferation.

In our BOE suppressor screens, *djc9* was the sole suppressor gene we obtained. The loss-of-function mutations of *djc9* render *asf1* dispensable for growth in unstressed conditions. Thus, the role of Asf1 needed for growth in fission yeast is to protect cells from a lethal effect exerted by Djc9. However, counteracting Djc9 is not the only function of Asf1, as *asf1Δ djc9Δ* double mutant exhibits strong DNA damage sensitivity, probably due to the loss of H3K56ac. It is noteworthy that Djc9 is evolutionarily conserved across eukaryotes, but intriguingly, it has been lost during evolution in a subset of budding yeast species, including the model organism *S. cerevisiae*, which may account for the non-essentiality of Asf1 in *S. cerevisiae* [11].

In fission yeast, Asf1 has been shown to function in the same pathway as the HIRA complex and the Clr6 histone deacetylase complex II to enforce heterochromatic silencing [82]. Interestingly, we found that the loss of Djc9 can suppress the heterochromatic silencing defect in the *asf1-1* mutant, as well as the growth defect and genotoxin sensitivity in the HIRA mutant *hip1Δ*, but not the growth defect in the Clr6 complex II mutant *alp13Δ* (Supplementary Fig. S7). These findings suggest that the role of Asf1 in heterochromatic silencing likely involves its antagonistic effect on Djc9-mediated histone degradation, while the HIRA complex functions alongside Asf1 to counteract Djc9.

We showed that human DNAJC9 can inhibit the growth of Asf1-depleted fission yeast cells in the absence of endogenous Djc9. This finding suggests that DNAJC9, like Djc9, can bind H3–H4 and promote their degradation in fission yeast. However, it remains unclear whether DNAJC9 facilitates histone degradation in human cells. While Djc9 is dispensable for cell viability in fission yeast, the DepMap database indicates that DNAJC9 is essential for cell viability in the majority of cancer cell lines [83]. Furthermore, knockout of *DNAJC9* in mice results in embryonic lethality, as reported by the International Mouse Phenotyping Consortium [84]. These observations suggest that DNAJC9 may play additional roles in mammals that are not shared by Djc9 in fission yeast, or that its conserved functions are more critical for viability in mammals.

Our analysis using Alphafold-Multimer revealed two different conformations of Djc9 and DNAJC9 when binding to histones. Functional analysis indicated that the α6-bound conformation but not the α0-bound conformation is functionally important. However, it is possible that both conformations exist in the cellular context. One possibility is that the α0-bound conformation and the α6-bound conformation correspond to the non-Hsp70-bound and the Hsp70-bound states, respectively, as α0 is located next to the J-domain and may be more susceptible to steric interference when the J-domain binds to Hsp70.

Histone chaperones are known to compete for histone binding [85]. For example, in metazoa, DAXX competes with Asf1 in binding histone H3.3 and promotes the deposition of H3.3 at heterochromatic loci [86]. In addition, DAXX also competes with HJURP in binding the centromeric histone variant CENP-A, and can lead to mislocalization of CENP-A when the function of HJURP is compromised [87, 88]. Our data support a competition model in which Asf1 competes with Djc9 for H3–H4 binding and thereby inhibits histone degradation mediated by Djc9. Notably, our *in vitro* competition assay showed that Asf1 possesses a higher affinity for H3–H4 compared to Djc9. During the normal cell cycle, Asf1 likely capitalizes on its superior affinity to minimize the extent of H3–H4-binding by Djc9. In the absence of Asf1, Djc9 is no longer restrained from binding H3–H4, and wreaks havoc by causing the degradation of H3–H4.

In contrast to the lethal effect in *asf1Δ* cells, when Asf1 is present, Djc9 plays a beneficial role in enhancing cell tolerance to HU treatment by promoting the degradation of newly synthesized histones that are in excess and overwhelm the capacity of Asf1 to protect them. It is still unclear how excess histones exert adverse effects in *S. pombe*. It has been proposed that in *S. cerevisiae*, the lack of the protease Wss1 results in the accumulation of single-stranded DNA-bound histones and thereby causes HU sensitivity [27, 89]. It is possible that the non-specific binding of excess histones to single-stranded DNA underlies the HU sensitivity of *djc9Δ* cells.

In addition to its role in promoting the degradation of endogenously accumulated free histones, we found that Djc9 confers protection against certain dysfunctional histone mutants by facilitating their degradation. Notably, loss-of-function missense mutations in H3 are dispersed throughout the H3 αN helix and the histone-fold [53], but mutations that render H3 specifically toxic to *djc9Δ* but not wild-type cells are predominantly located in or around the H3 αN helix. The H3 αN helix is crucial for histone-DNA interactions, histone-histone interactions, histone-histone chaperone interactions, and histone post-modifications [1, 90–93]). It is possible that H3 mutants with mutations in or near the H3 αN helix may assemble poorly with DNA or be more frequently evicted from chromatin, leading to their accumulation as free histones that are targeted for Djc9-mediated degradation.

Finally, it is noteworthy that *de novo* missense mutations in the *H3-3A* or *H3-3B* genes associated with dominant neurodevelopmental disorders include L48R, I51N, and Q55K, which are mutations affecting residues in the H3 αN helix [79, 94]. Here, we examined one of these mutants, H3-L48R, and found that its overexpression was toxic to *djc9Δ* cells but not to wild-type cells. Our findings may shed light on mechanisms that influence the severity of diseases caused by pathological histone mutations.

In summary, our results demonstrate the following: (1) Djc9 exerts its toxicity in the absence of Asf1 by promoting the degradation of histones; (2) Djc9 enhances cell tolerance to HU treatment by promoting the degradation of newly synthesized histones H3 and H4; (3) Djc9 protects cells from the toxicity exerted by certain dysfunctional histone H3 mutants by promoting their degradation.

## Supplementary Material

gkaf036_Supplemental_File

## Data Availability

The AlphaFold-Multimer-predicted structures used in this study have been deposited in the ModelArchive database (modelarchive.org) with the accession codes ma-f63vd (the Djc9–H3–H4 complex), ma-rvfoz (the Djc9(122-282)–H3–H4 complex), ma-7q9jt (the DNAJC9–H3.3–H4 complex), ma-qdw0k (the DNAJC9(108-260)–H3.3–H4 complex), and ma-akk0x (the Asf1–H3–H4 complex). Other data underlying this article are available in the article and in its online supplementary material.
