# Supplementary material for: The ortholog of human DNAJC9 promotes histone H3–H4 degradation and is counteracted by Asf1 in fission yeast
Source: Nucleic Acids Res. 2025 Jan 29;53(3):gkaf036. doi: 10.1093/nar/gkaf036 (PMC11775587; doi:10.1093/nar/gkaf036)
Supplement: gkaf036_Supplemental_File [file gkaf036_supplemental_file.pdf]

Supplementary Information

The ortholog of human DNAJC9 promotes histone H3–H4 degradation and is counteracted by Asf1 in fission yeast

Yan Ding, Jun Li, He-Li Jiang, Fang Suo, Guang-Can Shao, Xiao-Ran Zhang, Meng-Qiu Dong, Chao-Pei Liu, Rui-Ming Xu, Li-Lin Du

TABLE OF CONTENTS

Supplementary Figure S1 2
Supplementary Figure S2 4
Supplementary Figure S3 7
Supplementary Figure S4 9
Supplementary Figure S5 11
Supplementary Figure S6 13
Supplementary Figure S7 15
Supplementary Table S1 17
Supplementary Table S2 20

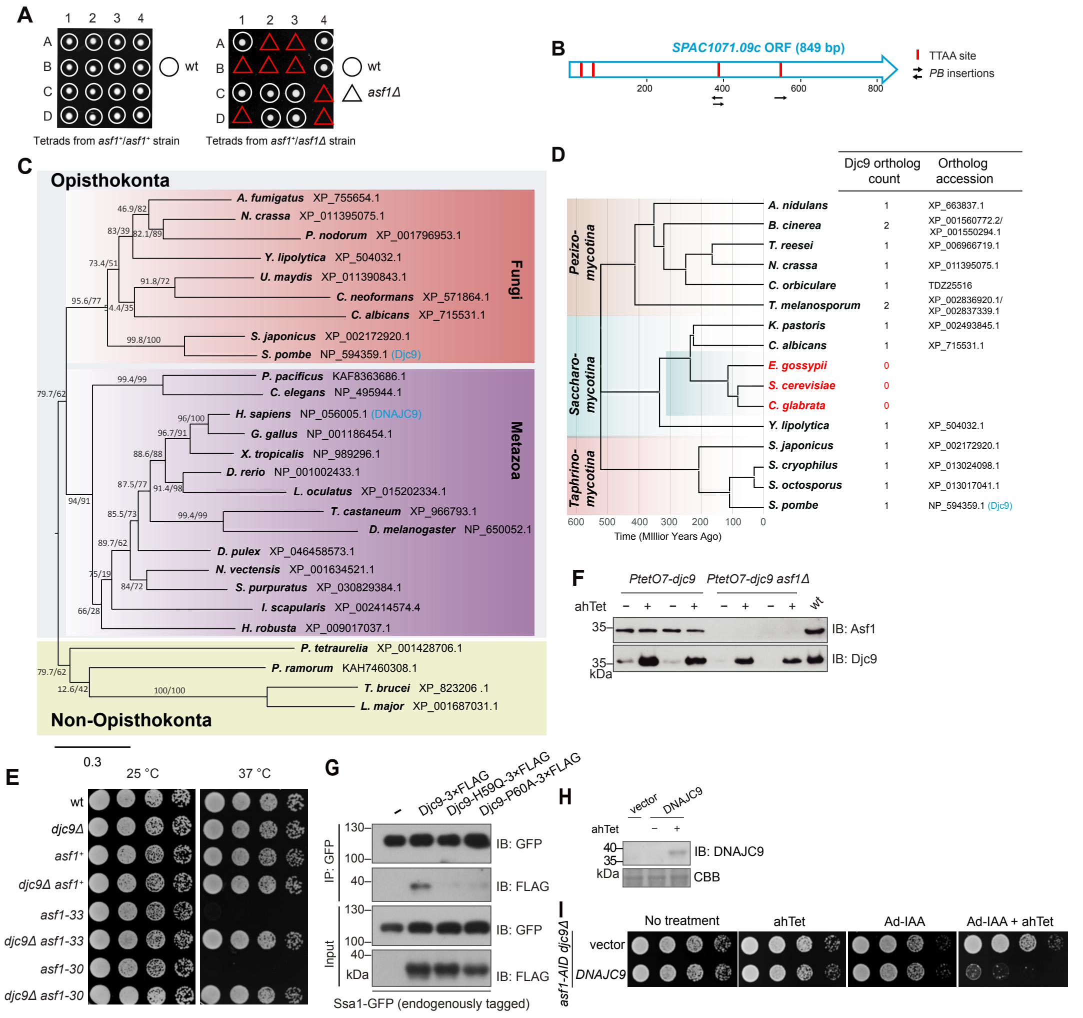

Supplementary Figure S1. In the absence of Djc9, *asf1* becomes dispensable for growth.

(A) *asf1* is an essential gene in fission yeast. Four tetrads from a wild-type (wt) diploid strain (the left panel) or from an *asf1+/asf1Δ* heterozygous diploid strain (the right panel) are shown. The four progeny from each of the tetrads are labeled as A, B, C, and D.

(B) Schematic of TTAA sites in *SPAC1071.09C/djc9* and the *PB* insertions observed in this gene when *PB*-insertion-induced BOE suppressor mutants of *asf1Δ* were analyzed by insertion junction sequencing. TTAA sites (denoted by red vertical line) are preferred insertion sites of *PB*. The orientation of *PB* insertion is indicated by black arrows. *PB* insertions of both forward and reverse orientations were observed at the third TTAA site,

and a *PB* insertion of the forward orientation was observed at the fourth TTAA site.
(C) Phylogenetic relationships of DNAJC9 homologs in representative eukaryotic species. A maximum likelihood tree was constructed using IQ-TREE and rooted using the proteins from non-Opisthokonta species as the outgroup. Branch labels represent the SH-aLRT support values (%) and the UFBoot support values (%) calculated by IQ-TREE. The scale bar indicates 0.3 substitutions per site.
(D) DNAJC9 homologs are absent in a subset of budding yeast species. Shown on the left is a time-calibrated phylogenetic tree of representative *Ascomycota* species. The number and accession(s) of DNAJC9 homolog(s) in each species are shown on the right. The species lacking DNAJC9 homologs are highlighted in red.
(E) The deletion of *djc9* suppressed the growth defects of temperature-sensitive *asf1* mutants at the restrictive temperature.
(F) The ahTet-induced expression of Djc9 was analyzed in a strain where the endogenous *djc9* gene is controlled by the *PtetO7* promoter. The expression level of Djc9 was analyzed by immunoblotting in the absence of ahTet or 5 h after the addition of ahTet. The induced expression level of Djc9 is similar to the expression level of Djc9 in a wild-type (wt) strain.
(G) Mutating the HPD motif of Djc9 disrupts the interaction between Djc9 and the HSP70 protein Ssa1. Ssa1 was endogenously tagged with GFP at the C-terminus. Djc9, tagged with FLAG at the C-terminus, was expressed under the *P41nmt1* promoter. Ssa1-GFP was immunoprecipitated using GBP beads, and the co-immunoprecipitated Djc9 was detected using an antibody against FLAG.
(H) The expression of human DNAJC9 under the promoter *PenotetSW2* was analyzed using an anti-DNAJC9 antibody.
(I) The expression of human DNAJC9 renders *asf1-AID djc9Δ* cells inviable. The expression of Djc9 and human DNAJC9 was controlled by the ahTet-inducible promoter *PenotetSW2*.

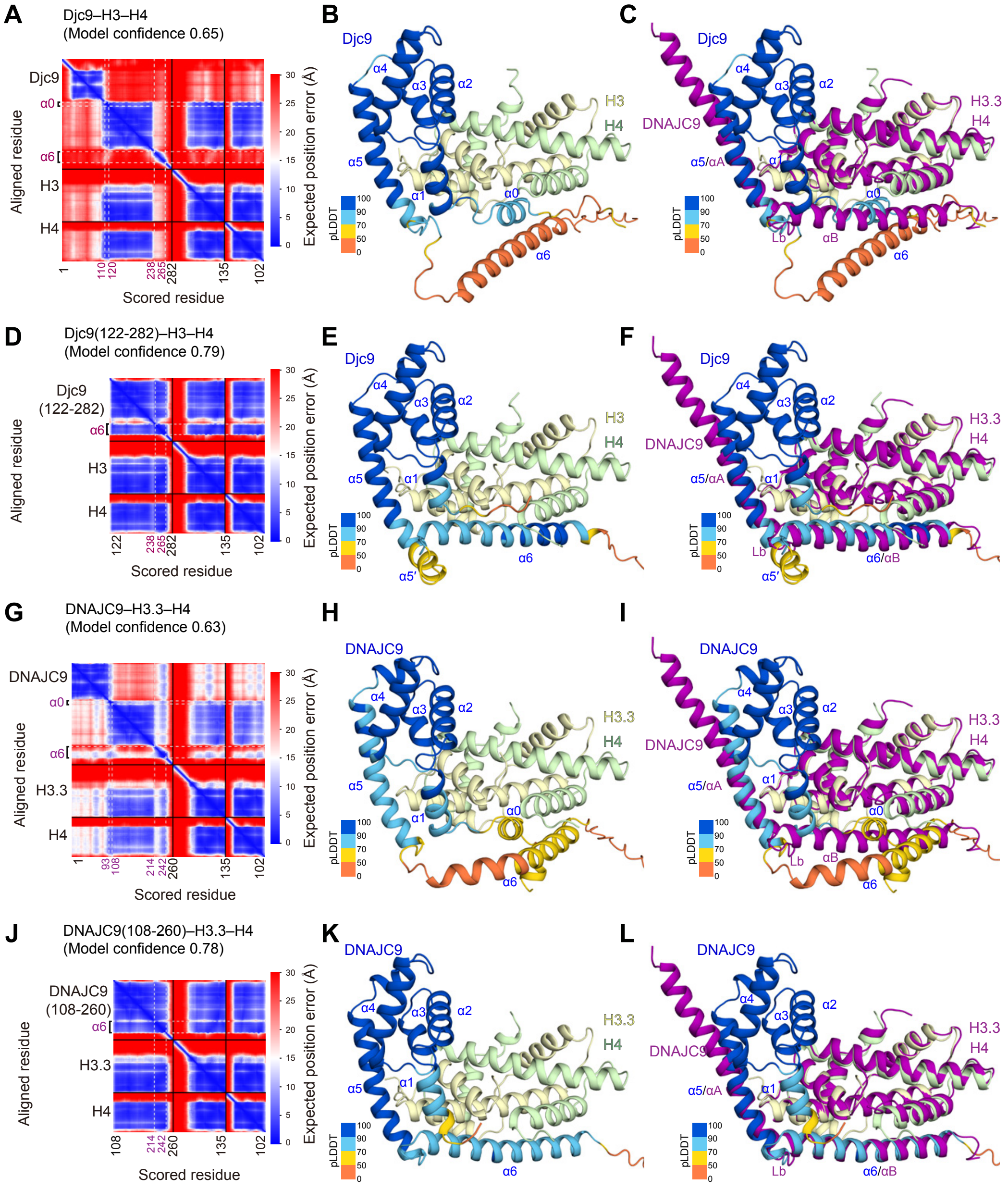

Supplementary Figure S2. AlphaFold-Multimer predicts two different histone-binding conformations of Djc9 and DNAJC9.

(A) Predicted aligned error (PAE) plot for the AlphaFold-Multimer-predicted structure of the Djc9–H3–H4 complex. The PAE is a confidence metric provided by AlphaFold, reflecting the expected error in the relative position of a pair of residues in the predicted structure, measured in Ångströms (Å). The PAE plot visualizes these values as a heatmap,

with the x- and y-coordinates representing residue numbers and the color in the heatmap indicating the PAE values. The color at the coordinates (x, y) denotes the expected error in the positioning of residue x when the predicted and true structures are aligned at residue y. Low PAE values (blue) indicate high confidence in relative residue positions, while high PAE values (red) suggest low confidence. Typically, residue pairs within the same domain, in interacting domains of the same protein, or in interacting domains of different proteins exhibit low PAE values. Black solid lines separate individual proteins, while white dashed lines highlight the Djc9 helices α0 and α6.

(B) Cartoon representation of the AlphaFold-Multimer-predicted structure of the Djc9–H3–H4 complex. For clarity, residues 1-99 of Djc9 encompassing the J-domain are not shown. Residues 100-282 of Djc9 are colored according to pLDDT scores, which are per-residue estimates of model confidence. pLDDT scores range from 0 to 100, with higher scores indicating greater confidence. A pLDDT score above 90 reflects extremely high confidence in the prediction, while scores between 70 and 90 indicate confident predictions. Scores between 50 and 70 reflect low confidence, and scores below 50 suggest a lack of confidence and indicate unstructured regions. H3 and H4 are colored pale yellow and pale green, respectively. The N-terminal tails of H3 and H4, predicted to be unstructured, are not shown.

(C) Structural comparison between the AlphaFold-Multimer-predicted structure of the Djc9–H3–H4 complex shown in (B) and the crystal structure of the human DNAJC9 histone-binding domain (HBD) in complex with H3.3–H4 (PDB: 7CJ0, purple). H3 in the predicted structure is superimposed on H3.3 in 7CJ0. The secondary structures in the DNAJC9 HBD, consisting of two α-helices (αA and αB) and a loop (Lb) in between, are named as described (66). αA and αB correspond to α5 and α6 in Djc9.

(D) PAE plot of the AlphaFold-Multimer-predicted structure of Djc9(122-282) in complex with H3–H4.

(E) Cartoon representation of the AlphaFold-Multimer-predicted structure of the Djc9(122-282)–H3–H4 complex. Coloring is as in (B).

(F) Structural comparison between the AlphaFold-Multimer-predicted structure of the

Djc9(122-282)–H3–H4 shown in (E) and the crystal structure of DNAJC9 HBD–H3.3–H4 complex (PDB 7CJ0, purple). H3 in the predicted structure is superimposed on H3.3 in 7CJ0.

(G) PAE plot of the AlphaFold-Multimer-predicted structure of the DNAJC9–H3.3–H4 complex.

(H) Cartoon representation of the AlphaFold-Multimer-predicted structure of the DNAJC9–H3.3–H4. For clarity, residues 1-86 of DNAJC9 encompassing the J-domain are not shown. Residues 87-260 of DNAJC9 are colored according to pLDDT scores. H3.3–H4 are colored pale yellow and pale green, respectively.

(I) Structural comparison between the AlphaFold-Multimer-predicted structure of the DNAJC9–H3.3–H4 shown in (H) and the crystal structure of DNAJC9 HBD–H3.3–H4 complex (PDB 7CJ0, purple). H3.3 in the predicted structure is superimposed on H3.3 in 7CJ0.

(J) PAE plot of the AlphaFold-Multimer-predicted structure of DNAJC9(108-260) in complex with H3.3–H4.

(K) Cartoon representation of the AlphaFold-Multimer-predicted structure of the DNAJC9(108-260)–H3.3–H4 complex. Coloring is as in (H).

(L) Structural comparison between the AlphaFold-Multimer-predicted structure of the DNAJC9–H3.3–H4 shown in (K) and the DNAJC9 HBD–H3.3–H4 complex (PDB 7CJ0, purple). H3.3 in the predicted structure is superimposed on H3.3 in 7CJ0.

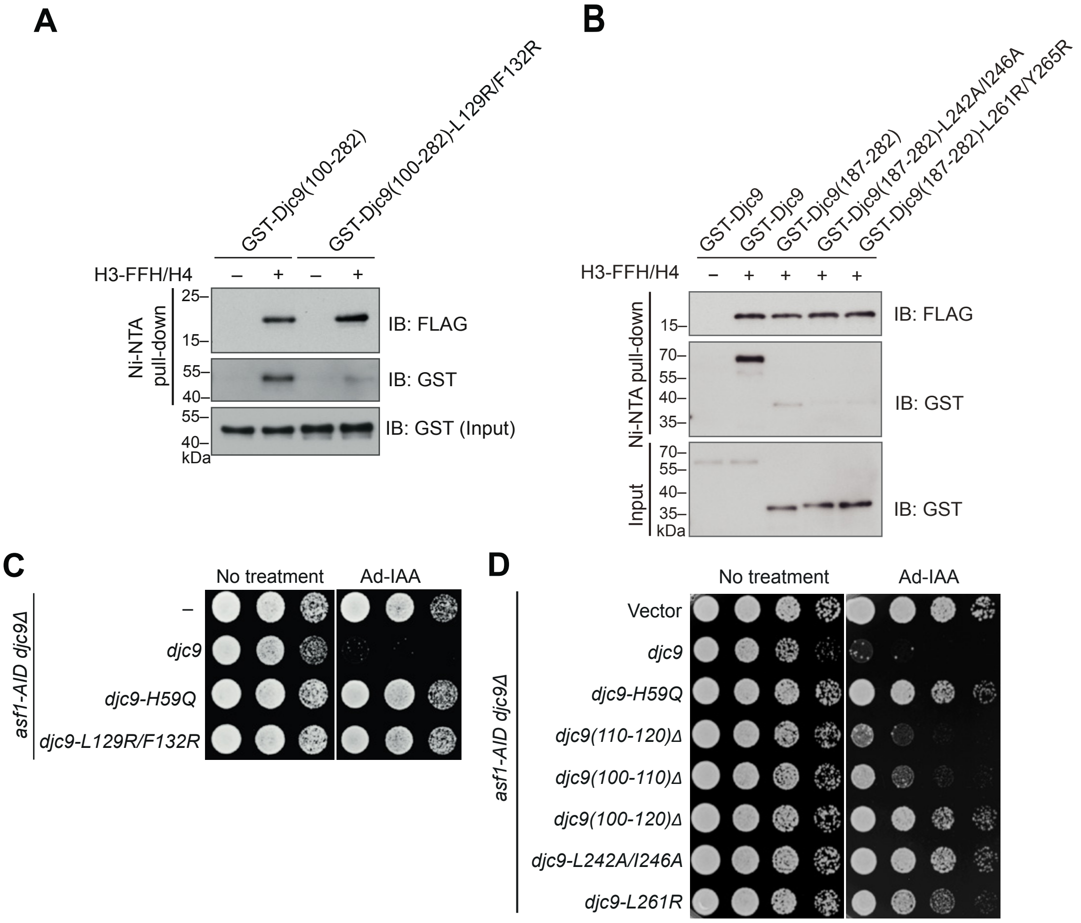

Supplementary Figure S3. The Djc9-H3 interaction is required for the toxicity of Djc9 to Asf1-deficient cells.

(A) Mutating Djc9 residues on the predicted interface between Djc9 and H3-α3 reduced histone binding. The GST-tagged C-terminal region (residues 100-282) of Djc9 carrying L129R/F132R mutations was co-expressed with FLAG2-His6 (FFH)-tagged H3 and untagged H4 in *E. coli*. Ni-NTA resin was used to pull down H3-FFH.

(B) Mutating Djc9 residues on the predicted interface between Djc9-α6 and H3-α2 also reduced histone binding. A GST-tagged C-terminal fragment of Djc9 (residues 187-282, corresponding to human DNAJC9 HBD) carrying L242A/I246A or L261R/Y265R mutations was co-expressed with FLAG2-His6 (FFH)-tagged H3 and untagged H4 in *E. coli*. Ni-NTA resin was used to pull down H3-FFH.

(C) The Djc9-L129R/F132R mutations abolished the toxicity of Djc9 to Asf1-depleted

cells. Wild-type or a mutant form of Djc9 under the native promoter was expressed in *asf1-AID djc9Δ* cells. Growth in the absence or presence of Ad-IAA was analyzed. Djc9-H59Q served as a loss-of-function control.

(D) The effects of deletion or mutation of helices α0 and α6 of Djc9 on its function in Asf1-depleted cells. Wild-type or a mutant form of Djc9 under the native promoter was expressed in *asf1-AID djc9Δ* cells. Growth in the absence or presence of Ad-IAA was analyzed. Djc9-H59Q served as a loss-of-function control.

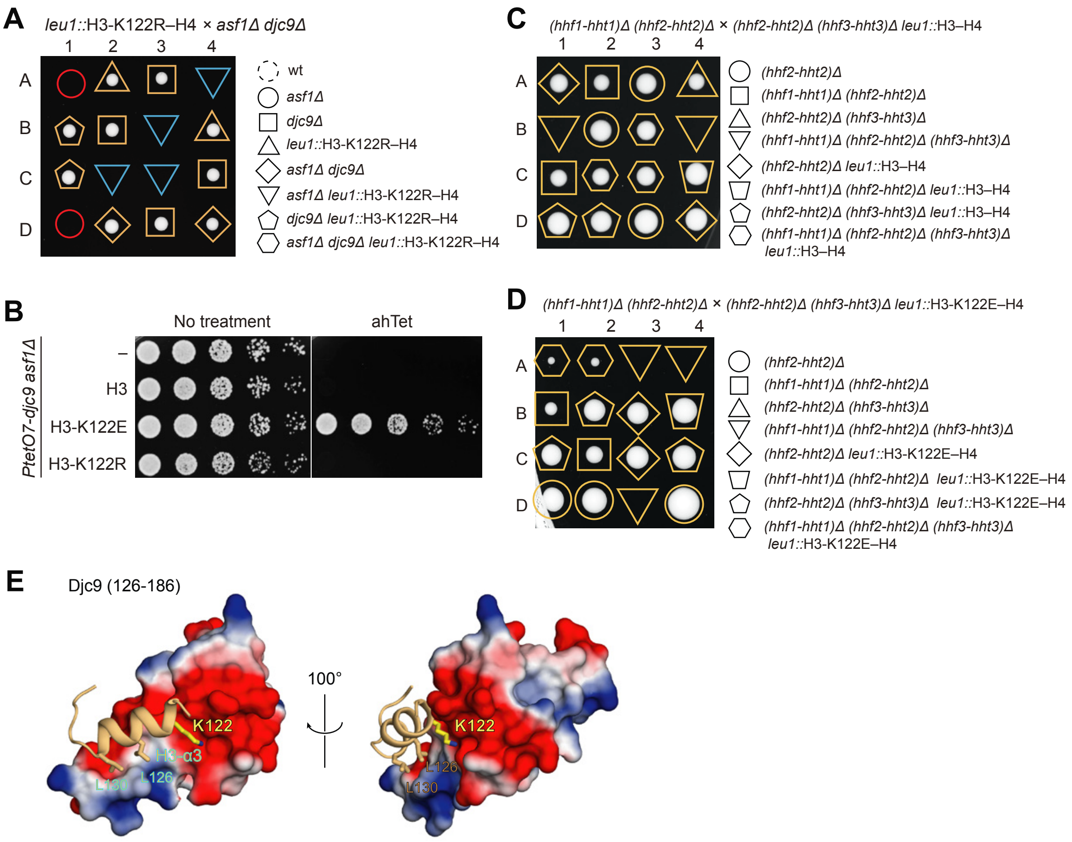

Supplementary Figure S4. H3-K122E can function as the only form of H3 in the cell.

(A) Tetrad analysis showing that H3-K122R could not suppress the lethality of *asf1Δ*. H3 and H4 were expressed from the same plasmid under the control of their native promoters.

(B) The expression of H3-K122E, but not H3-K122R, allowed *asf1Δ PtetO7-djc9* cells to grow when Djc9 expression was induced. H3 and H4 were expressed from the same plasmid under the control of their native promoters.

(C and D) Tetrad dissection showing that wild-type H3 (D) and H3-K122E (E) expressed from an integrating plasmid can support growth in the absence of endogenous H3.

(E) Close-up views showing that in the AlphaFold-Multimer-predicted structure of the Djc9–H3–H4 complex, the H3-K122 residue is in contact with an acidic surface of Djc9. Only residues 120-135 of H3 (light orange) and residues 126-186 of Djc9 (colored by

electrostatic potential) are shown. H3-K122 is depicted in stick representation and highlighted in yellow.

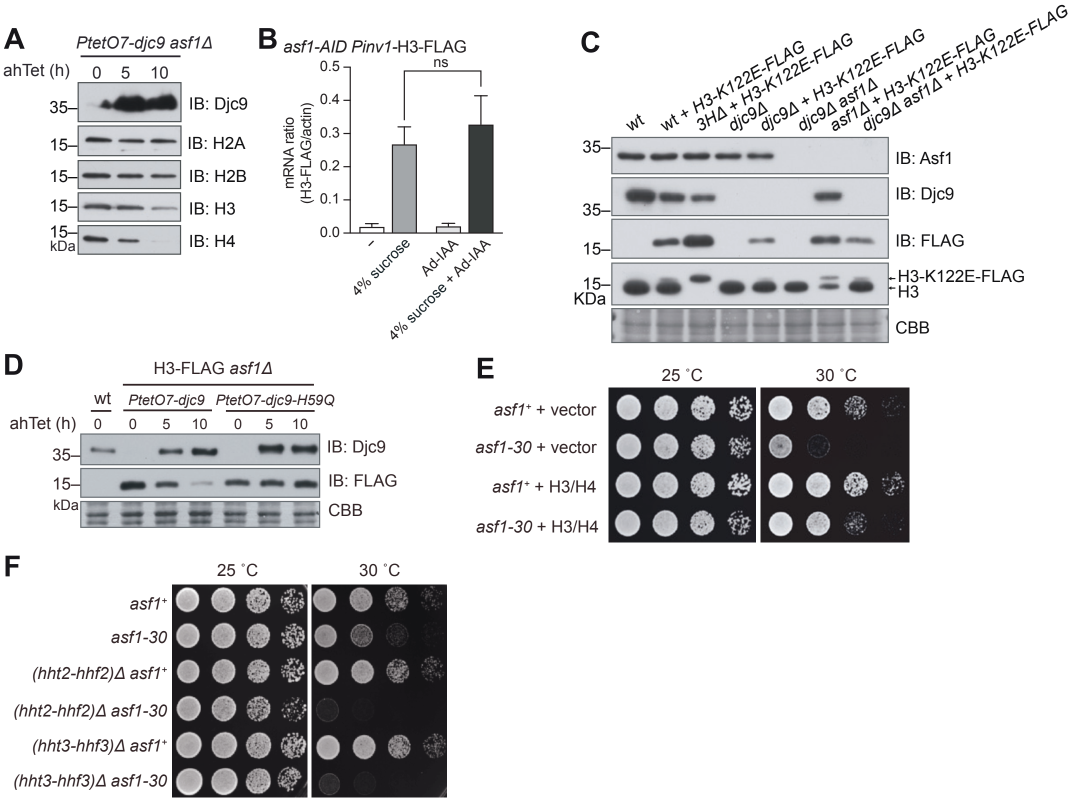

Supplementary Figure S5. Djc9-dependent H3–H4 degradation causes lethality of *asf1Δ* cells.

(A) Immunoblotting showing that inducing the expression of Djc9 in *PtetO7-djc9 asf1Δ* cells caused the reduction of the protein levels of H3 and H4 but not H2A and H2B. *PtetO7-djc9 asf1Δ* cells were treated with ahTet to induce Djc9 expression at the indicated time points and whole cell extracts were subjected to immunoblotting analysis.

(B) RT-qPCR analysis of the mRNA levels of H3-FLAG. *asf1-AID Pinv1-H3-FLAG* cells grown in 8% glucose medium were either shifted to 4% sucrose induction medium for 1 hour, treated with Ad-IAA in 8% glucose medium for 1 hour, or subjected to Ad-IAA treatment in 8% glucose medium for 1 hour followed by a shift to 4% sucrose induction medium with Ad-IAA for an additional 1 hour. Total RNA was isolated and RT-qPCR was performed using a pair of primers specific to H3-FLAG. The relative expression levels of H3-FLAG were normalized to actin mRNA levels. Three independent biological

replicates were performed. Error bars represent the standard deviation. The significance of fold changes was estimated using ordinary one-way ANOVA. ns indicates not significant.

(C) The expression levels of integrating-plasmid-expressed H3-K122E and endogenous H3 in wild-type, *3HΔ* (lacking endogenous histones), *djc9Δ*, *asf1Δ*, and *asf1Δ djc9Δ* backgrounds. C-terminally FLAG-tagged H3-K122E was expressed together with H4 under their native promoters in the indicated strains. The whole cell extracts were analyzed using immunoblotting with antibodies against FLAG and H3. The anti-H3 antibody recognizes both H3-FLAG and the endogenous H3.

(D) Djc9-mediated histone degradation depends on the HPD motif of the J-domain of Djc9. The expression of Djc9 or Djc9-H59Q under the *PtetO7* promoter was induced in the *asf1Δ* background. The levels of Djc9 and H3-FLAG were monitored by immunoblotting.

(E) Increasing the dosage of H3 and H4 genes mitigated the growth defect of a temperature-sensitive *asf1* mutant (the mutant allele is different from the one shown in Figure 5E).

(F) Decreasing the dosage of H3 and H4 genes exacerbated the growth defect of a temperature-sensitive *asf1* mutant (the mutant allele is different from the one shown in Figure 5F).

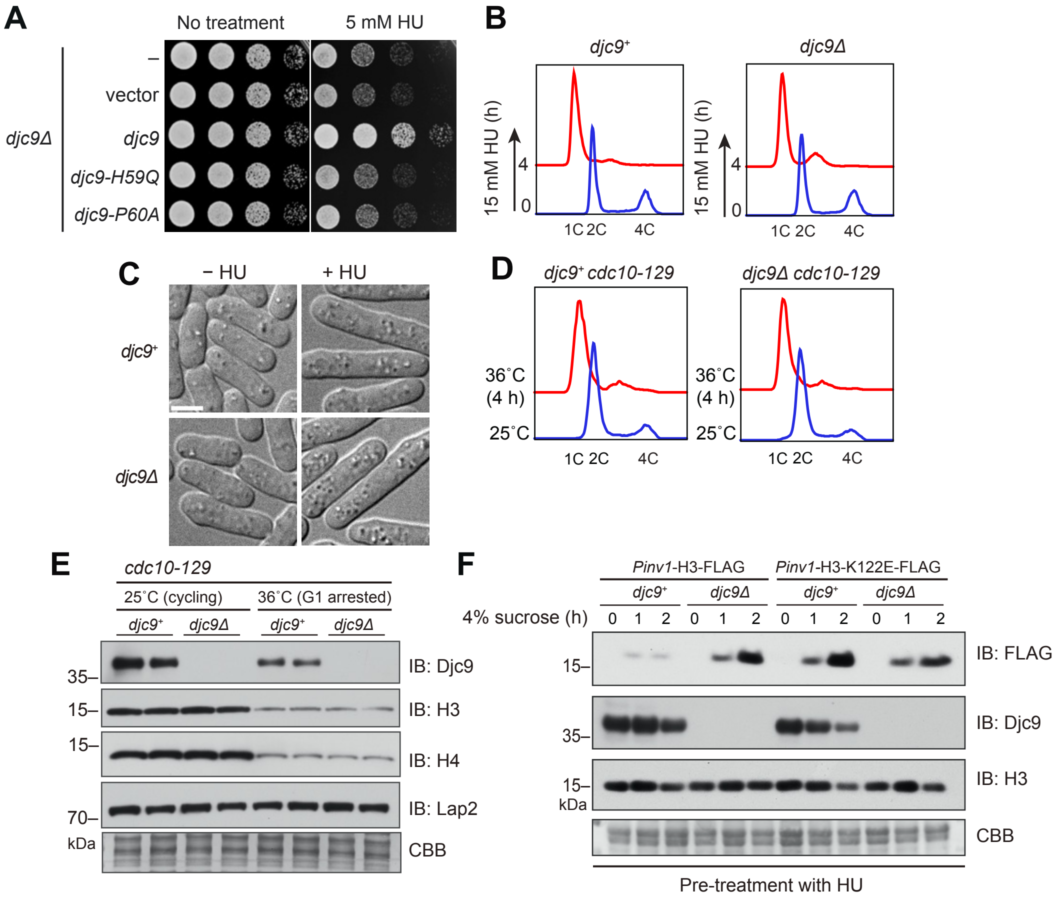

Supplementary Figure S6. Djc9-dependent H3–H4 degradation confers resistance to HU.

(A) The HPD motif is required for Djc9 to confer resistance to HU.

(B) FACS analysis showing the DNA content of asynchronous cells and cells arrested in early S phase by 15 mM HU.

(C) Micrographs showing that both wild-type and *djc9Δ* cells are elongated after HU treatment. DIC, differential interference contrast. Scale bar, 5 μm.

(D) FACS analysis showing the DNA content of asynchronous *cdc10-129* cells grown at a permissive temperature (25 °C) and *cdc10-129* cells arrested in the G1 phase (shifted to 36 °C for 4 h).

(E) Immunoblotting showing that the levels of H3 and H4 in G1-arrested *djc9Δ* cells are similar to those in G1-arrested wild-type cells.

(F) Immunoblotting showing that in HU-arrested cells, newly synthesized H3-K122E-FLAG, but not H3-FLAG, accumulates in the presence of Djc9.

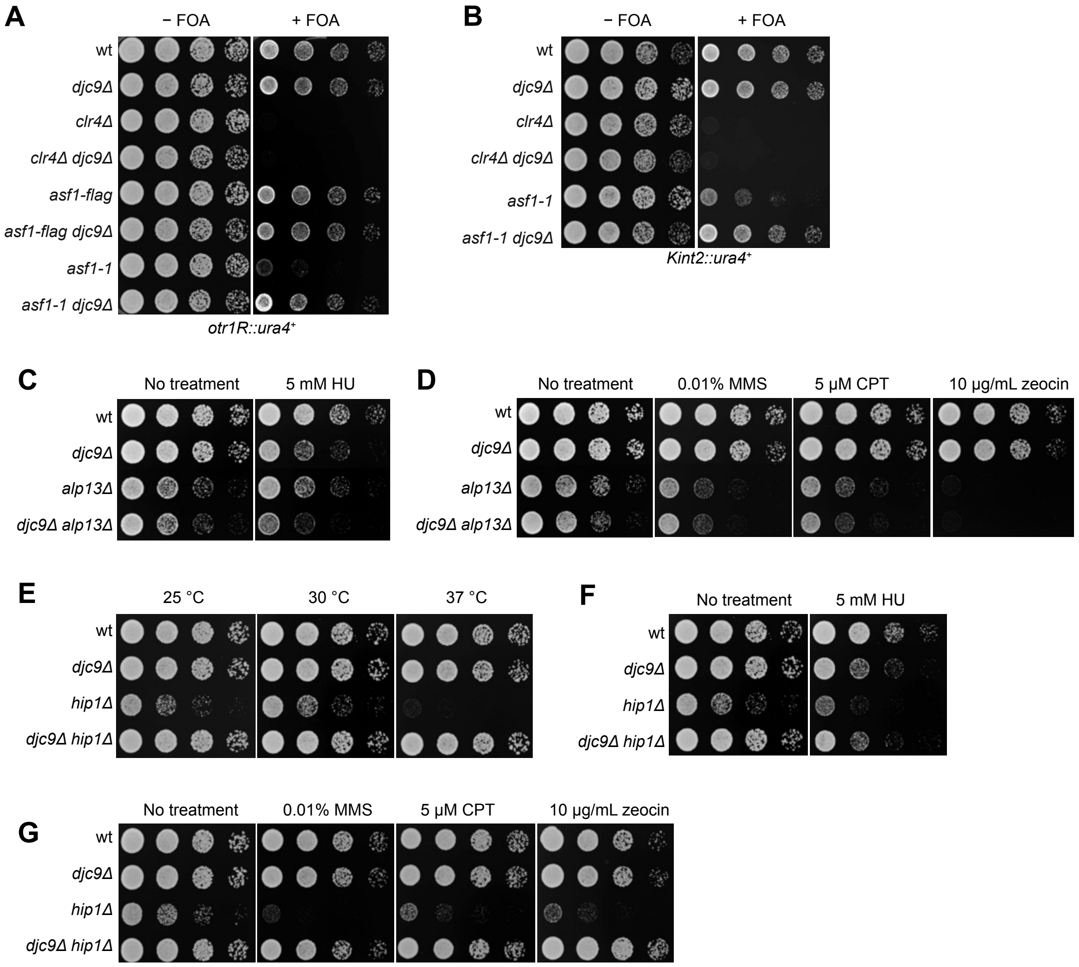

Supplementary Figure S7. The suppression effect of *djc9Δ* on the silencing defect of *asf1-1* and the growth defects and genotoxin sensitivity of *hip1Δ*.

(A and B) Loss of *djc9* suppressed the heterochromatin silencing defects in the *asf1-1* mutant. *djc9* was deleted in wild-type, *clr4Δ*, and *asf1-1* mutant strains carrying reporter genes at pericentromeric repeats (*otr1R*::*ura4*$^{+}$) in (A) and at the mating type donor region (*Kint2*::*ura4*$^{+}$) in (B). Five-fold serial dilutions of cells were spotted on counter-selective plates containing 5-fluoroorotic acid (FOA) to assay *ura4*$^{+}$ expression.

(C) The *djc9Δ alp13Δ* double deletion exhibited cumulative sensitivity to HU. Five-fold

serial dilutions of cells with the indicated genotypes were spotted on a YES plate and a YES plate containing HU.

(D) Loss of *djc9* did not alleviate the sensitivities of *alp13Δ* to genotoxic agents. Five-fold serial dilutions of cells with the indicated genotypes were spotted on a YES plate and YES plates containing MMS, CPT, or zeocin.

(E) Loss of *djc9* suppressed the growth defects of *hip1Δ*. Five-fold serial dilutions of cells with the indicated genotypes were spotted on YES plates and cultured under different temperature conditions.

(F and G) Loss of *djc9* suppressed the sensitivities of *hip1Δ* to genotoxic agents. Five-fold serial dilutions of cells with the indicated genotypes were spotted on a YES plate and YES plates containing HU (F), and on YES plates containing MMS, CPT, or zeocin (G).

Supplementary Table S1. Fission yeast strains used in this study

Strains	Genotypes	Related to
DY51760	*h+ his3-D1 leu1-32 ura4-D18*	
DY51761	*h− his3-D1 leu1-32 ura4-D18*	
DY51762	*h+ djc9Δ:: natMX his3-D1 leu1-32 ura4-D18*	
DY51763	*h− djc9Δ:: natMX his3-D1 leu1-32 ura4-D18*	
DY51764	*h− asf1Δ:: kanMX djc9Δ:: natMX his3-D1 leu1-32 ura4-D18*	Figureure 1B, 1E, 1F, 4A, 4B
DY51765	*h+ asf1-XTEN16-3×AID-spo5DSR:: kanMX ade6:: pAde6*^{PmeI}*-Padh1-atTIR*$^{F79A/D170E/M473L}$*:: bsdMX his3-D1 leu1-32 ura4-D18*	Figureure 1C
DY51766	*h+ djc9Δ:: natMX asf1-XTEN16-3×AID-spo5DSR:: kanMX ade6:: pAde6PmeI-Padh1-atTIR*$^{F79A/D170E/M473L}$*:: bsdMX his3-D1 leu1-32 ura4-D18*	Figureure 1C
DY51767	*h+ PtetO7-TATACYC1-djc9:: hphMX ura4-D18:: [tetR-tup11Δ70+ura4] leu1-32 ura4-D18*	Figureure 1D, 5B
DY51768	*h− asf1Δ:: kanMX PtetO7-TATACYC1-djc9:: hphMX ura4-D18:: [tetR-tup11Δ70+ura4] leu1-32 ura4-D18*	Figure 1D, 5B, S5A
DY51769	*h+ asf1-13×myc:: kanMX his3-D1 leu1-32 ura4-D18*	Figure S1E
DY51770	*h+ asf1-13×myc:: kanMX djc9Δ:: natMX his3-D1 leu1-32 ura4-D18*	Figure S1E
DY51771	*h+ asf1-30-13×myc:: kanMX his3-D1 leu1-32 ura4-D18*	Figure S1E
DY51772	*h+ asf1-30-13×myc:: kanMX djc9Δ:: natMX his3-D1 leu1-32 ura4-D18*	Figure S1E
DY51773	*h− asf1-33-13×myc:: kanMX his3-D1 leu1-32 ura4-D18*	Figure S1E
DY51774	*h− asf1-33-13×myc:: kanMX djc9Δ:: natMX his3-D1 leu1-32 ura4-D18*	Figure S1E
DY51775	*h+ his3-D1 leu1-32 ura4-D18 rtt109Δ:: kanMX*	Figure 1E, 1F
DY51776	*h+ his3-D1 leu1-32 ura4-D18 djc9Δ:: natMX asf1Δ:: hphMX*	Figure 1E, 1F
DY51777	*h+ his3-D1 leu1-32 ura4-D18 djc9Δ:: natMX rtt109Δ:: kanMX*	Figure 1E, 1F
DY51778	*h+ his3-D1 leu1-32 ura4-D18 djc9Δ:: natMX asf1Δ:: hphMX rtt109Δ:: kanMX*	Figure 1E, 1F
DY51779	*h− ade6:: pAde6*^{PmeI}*-Pdjc9-mCherry:: hphMX djc9Δ:: natMX asf1-XTEN16-3×AID-spo5DSR:: KanMX6 leu1-32:: Padh1-atTIR1*$^{F79A/D170E/M473L}$ *leu1-32 ura4-D18*	Figure 1G
DY51780	*h− ade6:: pAde6*^{PmeI}*-Pdjc9-djc9-mCherry:: hphMX djc9Δ:: natMX asf1-XTEN16-3×AID-spo5DSR:: KanMX6 leu1-32:: Padh1-atTIR1*$^{F79A/D170E/M473L}$ *leu1-32 ura4-D18*	Figure 1G
DY51781	*h− ade6:: pAde6*^{PmeI}*-Pdjc9-djc9-H59Q-mCherry:: hphMX djc9Δ:: natMX asf1-XTEN16-3×AID-spo5DSR:: KanMX6 leu1-32:: Padh1-atTIR1*$^{F79A/D170E/M473L}$ *leu1-32 ura4-D18*	Figure 1G
DY51782	*h− ade6:: pAde6*^{PmeI}*-Pdjc9-djc9-P60A-mCherry:: hphMX djc9Δ:: natMX asf1-XTEN16-3×AID-spo5DSR:: KanMX6 leu1-32:: Padh1-atTIR1*$^{F79A/D170E/M473L}$ *leu1-32 ura4-D18*	Figure 1G
DY51783	*h+ ssa1-GFP:: kanMX his3-D1 leu1-32 ura4-D18*	Figure S1G
DY51784	*h+ ssa1-GFP:: kanMX ade6:: pAde6*^{NotI}*-Pnmt41-djc9-3×FLAG:: hphMX his3-D1 leu1-32 ura4-D18*	Figure S1G
DY51785	*h+ ssa1-GFP:: kanMX ade6:: pAde6*^{NotI}*-Pnmt41-djc9-H59Q-3×FLAG-hphMX his3-D1 leu1-32 ura4-D18*	Figure S1G
DY51786	*h+ ssa1-GFP:: kanMX ade6:: pAde6*^{NotI}*-Pnmt41-djc9-P60A-3×FLAG:: hphMX his3-D1 leu1-32 ura4-D18*	Figure S1G
DY51787	*h+ ade6:: pAde6*$^{Not 1}$*-pCMV-tetR-PenotetSW2-hDNAJC9-FLAG:: hphMX djc9Δ:: natMX asf1-XTEN16-3×AID-spo5DSR:: KanMX6 leu1-32:: Padh1-atTIR1*$^{F79A/D170E/M473L}$ *leu1-32 ura4-D18*	Figure S1H, S1I
DY51788	*h+ leu1-32::nmt1-djc9-GFP djc9D::natMX his3-D1 leu1-32 ura4-D18*	Figure 2A
DY51789	*h− ade6:: pAde6*^{PmeI}*-Pdjc9-djc9-L129R/F132R-mCherry:: hphMX djc9Δ:: natMX asf1-XTEN16-3×AID-spo5DSR:: KanMX6 leu1-32:: Padh1-atTIR1*$^{F79A/D170E/M473L}$ *leu1-32 ura4-D18*	Figure S3C
DY51790	*h− ade6:: pAde6*^{PmeI}*-Pdjc9-djc9-(110-120)Δ-mCherry:: hphMX djc9Δ:: natMX asf1-XTEN16-3×AID-spo5DSR:: KanMX6 leu1-32:: Padh1-atTIR1*$^{F79A/D170E/M473L}$ *leu1-32 ura4-D18*	Figure S3D
DY51791	*h− ade6:: pAde6*^{PmeI}*-Pdjc9-djc9-(100-110)Δ-mCherry:: hphMX djc9Δ:: natMX asf1-XTEN16-3×AID-spo5DSR:: KanMX6 leu1-32:: Padh1-atTIR1*$^{F79A/D170E/M473L}$ *leu1-32 ura4-D18*	Figure S3D
DY51792	*h− ade6:: pAde6*^{PmeI}*-Pdjc9-djc9-(100-120)Δ-mCherry:: hphMX djc9Δ:: natMX asf1-XTEN16-3×AID-spo5DSR:: KanMX6 leu1-32:: Padh1-atTIR1*$^{F79A/D170E/M473L}$ *leu1-32 ura4-D18*	Figure S3D
DY51793	*h− ade6:: pAde6*^{PmeI}*-Pdjc9-djc9-L242A/I246A-mCherry:: hphMX djc9Δ:: natMX asf1-XTEN16-3×AID-spo5DSR:: KanMX6 leu1-32:: Padh1-atTIR1*$^{F79A/D170E/M473L}$ *leu1-32 ura4-D18*	Figure S3D
DY51794	*h− ade6:: pAde6*^{PmeI}*-Pdjc9-djc9-L261R-mCherry:: hphMX djc9Δ:: natMX asf1-XTEN16-3×AID-spo5DSR:: KanMX6 leu1-32:: Padh1-atTIR1*$^{F79A/D170E/M473L}$ *leu1-32 ura4-D18*	Figure S3D
DY51795	*h+ leu1-32:: pDUAL-Pasf1-YFH asf1-XTEN16-3×AID-spo5DSR:: kanMX ade6:: pAde6*^{PmeI}*-Padh1-atTIR*$^{F79A/D170E/M473L}$*:: bsdMX his3-D1 leu1-32 ura4-D18*	Figure 3I
DY51796	*h+ leu1-32:: pDUAL-Pasf1-asf1-YFH asf1-XTEN16-3×AID-spo5DSR:: kanMX ade6:: pAde6*^{PmeI}*-Padh1-atTIR*$^{F79A/D170E/M473L}$*:: bsdMX his3-D1 leu1-32 ura4-D18*	Figure 3I
DY51797	*h+ leu1-32:: pDUAL-Pasf1-asf1(1-161)-YFH asf1-XTEN16-3×AID-spo5DSR:: kanMX ade6:: pAde6*^{PmeI}*-Padh1-atTIR*$^{F79A/D170E/M473L}$*:: bsdMX his3-D1 leu1-32 ura4-D18*	Figure 3I
DY51798	*h+ leu1-32:: pDUAL-Pasf1-asf1-V95R-YFH asf1-XTEN16-3×AID-spo5DSR:: kanMX ade6:: pAde6*^{PmeI}*-Padh1-atTIR*$^{F79A/D170E/M473L}$*:: bsdMX his3-D1 leu1-32 ura4-D18*	Figure 3I
DY51799	*h+ leu1-32:: pDUAL-hhf2-hht2-FLAG his3-D1 leu1-32 ura4-D18*	Figure 4B
DY51800	*h+ leu1-32:: pDUAL-hhf2-hht2-K122E-FLAG his3-D1 leu1-32 ura4-D18*	Figure 4A
DY51801	*h+ leu1-32:: pDUAL-hhf2-hht2-K122R-FLAG his3-D1 leu1-32 ura4-D18*	Figure S4A

DY51802	h− leu1-32:: pDUAL-FLAG-hhf2-hht2 (hhf2-hht2)Δ:: hphMX (hhf3-hht3)Δ:: nathMX his3-D1 leu1-32 ura4-D18	Figure S4C, tetrad analysis
DY51803	h− leu1-32:: pDUAL-FLAG-hhf2-hht2-K122E (hhf2-hht2)Δ:: hphMX (hhf3-hht3)Δ:: nathMX his3-D1 leu1-32 ura4-D18	Figure S4D, tetrad analysis
DY51804	h+ (hhf1-hht1)Δ:: kanMX (hhf2-hht2)Δ:: hphMX his3-D1 leu1-32 ura4-D18	Figure S4C, S4D, tetrad analysis
DY51805	h+ leu1-32:: pDUAL-hhf2-hht2-FLAG asf1Δ:: kanMX PtetO7-TATACYC1-djc9:: hphMX ura4-D18:: [tetR-tup11Δ70+ura4] leu1-32 ura4-D18	Figure S4B, 5D, S5D
DY51806	h+ leu1-32:: pDUAL-hhf2-hht2-K122E-FLAG asf1Δ:: kanMX PtetO7-TATACYC1-djc9:: hphMX ura4-D18:: [tetR-tup11Δ70+ura4] leu1-32 ura4-D18	Figure S4B, 5D
DY51807	h+ leu1-32:: pDUAL-hhf2-hht2-K122R-FLAG asf1Δ:: kanMX PtetO7-TATACYC1-djc9:: hphMX ura4-D18:: [tetR-tup11Δ70+ura4] leu1-32 ura4-D18	Figure S4B, 5D
DY51808	h+ asf1-13×myc:: kanMX djc9-TAP:: hphMX leu1-32:: pDUAL-hhf2-hht2-FLAG leu1-32 ura4-D18	Figure 4C, 4D
DY51809	h+ asf1-13×myc:: kanMX djc9-TAP:: hphMX leu1-32:: pDUAL-hhf2-hht2-K122E-FLAG leu1-32 ura4-D18	Figure 4C, 4D
DY51810	h+ asf1-13×myc:: kanMX djc9-TAP:: hphMX leu1-32:: pDUAL-hhf2-hht2-K122R-FLAG leu1-32 ura4-D18	Figure 4C, 4D
DY51811	h− leu1-32::pDUAL-hhf2-hht2-K122E-FLAG his3-D1 leu1-32 ura4-D18	Figure S5C
DY51812	h− leu1-32:: pDUAL-hhf2-hht2-K122E-FLAG g (hhf1-hht1)Δ::kanMX (hhf2-hht2)Δ::hphMX (hhf3-hht3)Δ::nathMX his3-D1 leu1-32 ura4-D18	Figure S5C
DY51813	h− leu1-32::pDUAL-hhf2-hht2-K122E-FLAG djc9Δ:: natMX his3-D1 leu1-32 ura4-D18	Figure S5C
DY51814	h+ leu1-32::pDUAL-hhf2-hht2-K122E-FLAG asf1Δ::kanMX his3-D1? leu1-32 ura4-D18	Figure S5C
DY51815	h+ leu1-32::pDUAL-hhf2-hht2-K122E-FLAG asf1Δ::kanMX djc9Δ:: natMX his3-D1? leu1-32 ura4-D18	Figure S5C
DY51816	h− asf1-XTEN16-3×AID-spo5DSR:: kanMX leu1-32:: pDUAL-Padh1-atTIR$^{F79A/D170E/M473L}$:: bsdMX leu1-32 ura4-D18	Figure 5A
DY51817	h− djc9Δ:: natMX asf1-XTEN16-3×AID-spo5DSR:: kanMX leu1-32:: pDUAL-Padh1-atTIR$^{F79A/D170E/M473L}$:: bsdMX leu1-32 ura4-D18	Figure 5A
DY51818	h+ leu1-32:: pDUAL-Pinv1-H3-FLAG asf1-XTEN16-3×AID:: kanMX ade6:: pAde6^{PmeI}-Padh1-atTIR$^{F79A/D170E/M473L}$:: bsdMX his3-D1 leu1-32 ura4-D18	Figure 5C
DY51819	h+ leu1-32:: pDUAL-Pinv1-H3-FLAG djc9Δ:: natMX asf1-XTEN16-3×AID:: kanMX ade6:: pAde6^{PmeI}-Padh1-atTIR$^{F79A/D170E/M473L}$:: bsdMX leu1-32 ura4-D18	Figure 5C
DY51820	h− leu1-32:: pDUAL-hhf2-hht2-FLAG asf1Δ:: kanMX PtetO7-TATACYC1-djc9-H59Q:: hphMX ura4-D18:: [tetR-tup11Δ70+ura4] leu1-32 ura4-D18	Figure S5D
DY51821	h− asf1-13×myc:: kanMX leu1-32:: pDUAL-FLAG -hhf2-hht2 his3-D1 leu1-32 ura4-D18	Figure 5E, S5E
DY51822	h+ asf1-30-13×myc:: kanMX leu1-32:: pDUAL-FLAG -hhf2-hht2 his3-D1 leu1-32 ura4-D18	Figure S5E
DY51823	h+ asf1-33-13×myc:: kanMX leu1-32:: pDUAL-FLAG -hhf2-hht2 his3-D1 leu1-32 ura4-D18	Figure 5E
DY51824	h? asf1-13×myc:: kanMX (hht2-hhf2)Δ:: hphMX his3-D1 leu1-32 ura4-D18	Figure 5F, S5F
DY51825	h? asf1-30-13×myc:: kanMX (hht2-hhf2)Δ:: hphMX his3-D1 leu1-32 ura4-D18	Figure S5F
DY51826	h? asf1-33-13×myc:: kanMX (hht2-hhf2)Δ:: hphMX his3-D1 leu1-32 ura4-D18	Figure 5F
DY51827	h? asf1-13×myc:: kanMX (hht3-hhf3)Δ:: natMX his3-D1 leu1-32 ura4-D18	Figure 5F, S5F
DY51828	h? asf1-30-13×myc:: kanMX (hht3-hhf3)Δ:: natMX his3-D1 leu1-32 ura4-D18	Figure S5F
DY51829	h? asf1-33-13×myc:: kanMX (hht3-hhf3)Δ:: natMX his3-D1 leu1-32 ura4-D18	Figure 5F
DY51830	h+ ade6:: pAde6^{NotI}-Pinv1-H3-FLAG:: hphMX his3-D1 leu1-32 ura4-D18	Figure 6D
DY51831	h+ ade6:: pAde6^{NotI}-Pinv1-H3-FLAG:: hphMX djc9Δ:: natMX his3-D1 leu1-32 ura4-D18	Figure 6D
DY51832	h+ (hht1-hhf1)Δ:: kanMX his3-D1 leu1-32 ura4-D18	Figure 6E
DY51833	h+ (hht1-hhf1)Δ:: kanMX djc9Δ:: natMX leu1-32 ura4-D18	Figure 6E
DY51834	h+ (hht2-hhf2)Δ:: hphMX his3-D1 leu1-32 ura4-D18	Figure 6E
DY51835	h+ (hht2-hhf2)Δ::hphMX djc9Δ:: natMX his3-D1 leu1-32 ura4-D18	Figure 6E
DY51836	h+ (hht3-hhf3)Δ:: bsdMX his3-D1 leu1-32 ura4-D18	Figure 6E
DY51837	h+ (hht3-hhf3)Δ:: bsdMX djc9Δ:: natMX his3-D1 leu1-32 ura4-D18	Figure 6E
DY51838	h+ djc9Δ:: natMX ade6:: pAde6^{PmeI}-Pdjc9-mcherry:: hphMX his3-D1 leu1-32 ura4-D18	Figure S6A
DY51839	h+ djc9Δ:: natMX ade6:: pAde6^{PmeI}- Pdjc9-djc9-mcherry:: hphMX his3-D1 leu1-32 ura4-D18	Figure S6A
DY51840	h+ djc9Δ:: natMX ade6:: pAde6^{PmeI}- Pdjc9-djc9-H59Q-mcherry:: hphMX his3-D1 leu1-32 ura4-D18	Figure S6A
DY51841	h+ djc9Δ:: natMX ade6:: pAde6^{PmeI}- Pdjc9-djc9-P60A-mcherry:: hphMX his3-D1 leu1-32 ura4-D18	Figure S6A
DY51842	h+ ade6:: pAde6^{NotI}-Pinv1-H3-K122E-FLAG:: hphMX his3-D1 leu1-32 ura4-D18	Figure S6F
DY51843	h+ ade6:: pAde6^{NotI}-Pinv1-H3-K122E-FLAG:: hphMX djc9Δ:: natMX his3-D1 leu1-32 ura4-D18	Figure S6F
DY51844	h? cdc10-129 his3-D1 leu1-32 ura4-D18	Figure S6D, S6E
DY51845	h? cdc10-129 djc9Δ:: natMX his3-D1 leu1-32 ura4-D18	Figure S6D, S6E
DY51846	h+ leu1-32:: pDUAL-Pnmt1-H3-GFP his3-D1 leu1-32 ura4-D18	Figure 7A–C
DY51847	h+ leu1-32:: pDUAL-Pnmt1-H3-GFP djc9Δ:: natMX his3-D1 leu1-32 ura4-D18	Figure 7A–C
DY51848	h+ his3-D1 leu1-32 ura4-D18 leu1-32:: pDUAL-Pnmt1-FLAG	Figure 7D–E
DY51849	h+ his3-D1 leu1-32 ura4-D18 leu1-32:: pDUAL-Pnmt1-H3-FLAG	Figure 7D–E
DY51850	h+ his3-D1 leu1-32 ura4-D18 leu1-32:: pDUAL-Pnmt1-H3-Y41A-FLAG	Figure 7D–E
DY51851	h+ his3-D1 leu1-32 ura4-D18 leu1-32:: pDUAL-Pnmt1-H3-Y41E-FLAG	Figure 7D–E

DY51852	*h+ his3-D1 leu1-32 ura4-D18 leu1-32:: pDUAL-Pnmt1-H3-L48A-FLAG*	Figure 7D–E
DY51853	*h+ his3-D1 leu1-32 ura4-D18 leu1-32:: pDUAL-Pnmt1-H3-L48R-FLAG*	Figure 7D–E
DY51854	*h+ his3-D1 leu1-32 ura4-D18 leu1-32:: pDUAL-Pnmt1-H3-I51A-FLAG*	Figure 7D–E
DY51855	*h+ his3-D1 leu1-32 ura4-D18 leu1-32:: pDUAL-Pnmt1 -H3-Q55A-FLAG*	Figure 7D–E
DY51856	*h+ his3-D1 leu1-32 ura4-D18 leu1-32:: pDUAL-Pnmt1-FLAG djc9Δ:: natMX*	Figure 7D–E
DY51857	*h+ his3-D1 leu1-32 ura4-D18 leu1-32:: pDUAL-Pnmt1-H3-FLAG djc9Δ:: natMX*	Figure 7D–E
DY51858	*h+ his3-D1 leu1-32 ura4-D18 leu1-32:: pDUAL-Pnmt1-H3-Y41A-FLAG djc9Δ:: natMX*	Figure 7D–E
DY51859	*h+ his3-D1 leu1-32 ura4-D18 leu1-32:: pDUAL-Pnmt1-H3-Y41E-FLAG g djc9Δ:: natMX*	Figure 7D–E
DY51860	*h+ his3-D1 leu1-32 ura4-D18 leu1-32:: pDUAL-Pnmt1-H3-L48A-FLAG djc9Δ:: natMX*	Figure 7D–E
DY51861	*h+ his3-D1 leu1-32 ura4-D18 leu1-32:: pDUAL-Pnmt1-H3-L48R-FLAG djc9Δ:: natMX*	Figure 7D–E
DY51862	*h+ his3-D1 leu1-32 ura4-D18 leu1-32:: pDUAL-Pnmt1-H3-I51A-FLAG djc9Δ:: natMX*	Figure 7D–E
DY51863	*h+ his3-D1 leu1-32 ura4-D18 leu1-32:: pDUAL-Pnmt1-H3-Q55A-FLAG djc9Δ:: natMX*	Figure 7D–E
DY51864	*h+ his3-D1 leu1-32 ura4-D18 leu1-32:: pDUAL-Pnmt1-H3-K122E-FLAG*	Figure 7F–G
DY51865	*h+ his3-D1 leu1-32 ura4-D18 leu1-32:: pDUAL-Pnmt1-H3-K122E/L48A-FLAG*	Figure 7F–G
DY51866	*h+ his3-D1 leu1-32 ura4-D18 leu1-32:: pDUAL-Pnmt1-H3-K122E/I51A-FLAG*	Figure 7F–G
DY51867	*h+ his3-D1 leu1-32 ura4-D18 leu1-32:: pDUAL-Pnmt1-H3-K122E/Y41A-FLAG*	Figure 7F–G
DY51868	*h+ his3-D1 leu1-32 ura4-D18 leu1-32:: pDUAL-Pnmt1-H3-K122E/Q55A-FLAG*	Figure 7F–G
DY51869	*h+ his3-D1 leu1-32 ura4-D18 leu1-32:: pDUAL-Pnmt1-H3-K122E/Y41E-FLAG*	Figure 7F–G
DY51870	*h+ his3-D1 leu1-32 ura4-D18 leu1-32:: pDUAL-Pnmt1-H3-K122E-FLAG djc9Δ:: natMX*	Figure 7F–G
DY51871	*h+ his3-D1 leu1-32 ura4-D18 leu1-32:: pDUAL-Pnmt1-H3-K122E/L48A-FLAG djc9Δ:: natMX*	Figure 7F–G
DY51872	*h+ his3-D1 leu1-32 ura4-D18 leu1-32:: pDUAL-Pnmt1-H3-K122E/I51A-FLAG djc9Δ:: natMX*	Figure 7F–G
DY51873	*h+ his3-D1 leu1-32 ura4-D18 leu1-32:: pDUAL-Pnmt1-H3-K122E/Y41A-FLAG djc9Δ:: natMX*	Figure 7F–G
DY51874	*h+ his3-D1 leu1-32 ura4-D18 leu1-32:: pDUAL-Pnmt1-H3-K122E/Q55A-FLAG djc9Δ:: natMX*	Figure 7F–G
DY51875	*h+ his3-D1 leu1-32 ura4-D18 leu1-32:: pDUAL-Pnmt1-H3-K122E/Y41E-FLAG djc9Δ:: natMX*	Figure 7F–G
DY51884	*Msmt0 leu1-32 his2 ade6-210 ura4 DS/E otr1R::ura4+*	Figure S7A
DY51885	*Msmt0 djc9Δ::hphMX leu1-32 his2 ade6-210 ura4 DS/E otr1R::ura4+*	Figure S7A
DY51886	*mat1msto ade6-216 ura4 DS/E leu1-32 his2 OtrR1::ura4 clr4D::KanMX*	Figure S7A
DY51887	*mat1msto djc9Δ::hphMX ade6-216 ura4 DS/E leu1-32 his2 OtrR1::ura4 clr4D::KanMX*	Figure S7A
DY51888	*h+ leu1-32 ade6-210 ura4 DS/E otr1R::ura4+ asf1-FLAG::Kan*	Figure S7A
DY51889	*h+ djc9Δ::hphMX leu1-32 ade6-210 ura4 DS/E otr1R::ura4+ asf1-FLAG::Kan*	Figure S7A
DY51890	*Msmt0 leu1-32 ade6-216 his2 otr1R::ura4+ asf1-1-FLAG::Kan*	Figure S7A
DY51891	*Msmt0 djc9Δ::hphMX leu1-32 ade6-216 his2 otr1R::ura4+ asf1-1-FLAG::Kan*	Figure S7A
DY51892	*h90 kint2::ura4+ ade6-210 ura4 DS/E leu1-32 his2*	Figure S7B
DY51893	*h90 djc9Δ::hphMX kint2::ura4+ ade6-210 ura4 DS/E leu1-32 his2*	Figure S7B
DY51894	*h90 kint2::ura4+ ade6-216 ura4 DS/E leu1-32 his2 clr4D::KanMX*	Figure S7B
DY51895	*h90 djc9Δ::hphMX kint2::ura4+ ade6-216 ura4 DS/E leu1-32 his2 clr4D::KanMX*	Figure S7B
DY51896	*h90 leu1-32 ade6-210 his2 kint2::ura4+ asf1-1-FLAG::kan*	Figure S7B
DY51897	*h90 djc9Δ::hphMX leu1-32 ade6-210 his2 kint2::ura4+ asf1-1-FLAG::kan*	Figure S7B
DY51898	*apl13Δ::hphMX his3-D1 leu1-32 ura4-D18*	Figure S7C, S7D
DY51899	*apl13Δ::hphMX djc9Δ::natMX his3-D1 leu1-32 ura4-D18*	Figure S7C, S7D
DY51900	*hip1Δ::hphMX his3-D1 leu1-32 ura4-D18*	Figure S7E, S7F, S7G
DY51901	*hip1Δ::hphMX djc9Δ::natMX his3-D1 leu1-32 ura4-D18*	Figure S7E, S7F, S7G

Supplementary Table S2. Plasmids used in this study

Plasmid ID	Plasmid Names	Related to
DB5763	pAde6^{PmeI}-Pdjc9-mCherry[hphMX]	Figure 1G
DB5764	pAde6^{PmeI}-Pdjc9-Djc9-mCherry[hphMX]	Figure 1G
DB5765	pAde6^{PmeI}-Pdjc9-Djc9-H59Q-mCherry[hphMX]	Figure 1G
DB5766	pAde6^{PmeI}-Pdjc9-Djc9-P60A-mCherry[hphMX]	Figure 1G
DB5767	paAde6^{NotI}-pDUAL-nmt41-Djc9-3×FLAG[hphMX]	Figure S1G
DB5768	paAde6^{NotI}-pDUAL-nmt41-Djc9-H59Q-3×FLAG[hphMX]	Figure S1G
DB5769	paAde6^{NotI}-pDUAL-nmt41-Djc9-P60A-3×FLAG[hphMX]	Figure S1G
DB5770	pAde6^{NotI}-PCMV-tetR-PenotetSW2-hDNAJC9-FLAG[hphMX]	Figure S1H, S1I
DB5771	pDUAL-Pnmt1-Djc9-GFP	Figure 2A
DB5772	pETDut-His$_6$-GST-(HRV-3C)-Djc9	Figure 3H
DB5773	pCDFDuet-1-H3–H4	Figure 2B, 3G, 3H
DB5774	pGEX4T-1-GST-Djc9	Figure 2B, 2E, 3G, 4E
DB5775	pCDFDuet-1-H3-FLAG$_2$-His$_6$–H4	Figure 2E, S3A, S3B, 3F, 4E
DB5776	pCDFDuet-1-H3-K122E-FLAG$_2$-His$_6$–H4.1	Figure 4E
DB5777	pGEX4T-1-GST-Djc9(1-99)	Figure 2E
DB5778	pGEX4T-1-GST-Djc9(100-282)	Figure 2E, S3A
DB5779	pGEX4T-1-GST-Djc9(122-282)	Figure 2E
DB5780	pGEX4T-1-GST-Djc9(122-195)	Figure 2E
DB5781	pGEX4T-1-GST-Djc9(196-282)	Figure 2E
DB5782	pGEX4T-1-GST-Djc9(122-186)	Figure 2E
DB5783	pGEX4T-1-GST-Djc9(187-282)	Figure 2E, S3B
DB5784	pGEX4T-1-GST-Djc9(100-282)-L129R/F132R	Figure S3A
DB5785	pGEX4T-1-GST-Djc9(187-282)-L241A/I246A	Figure S3B
DB5786	pGEX4T-1-GST-Djc9(187-282)-L261R/Y265R	Figure S3B
DB5787	pAde6^{PmeI}-Pdjc9-Djc9-L129R/F132R-mCherry[hphMX]	Figure S3C
DB5788	pAde6^{PmeI}-Pdjc9-Djc9-(110-120)Δ-mCherry[hphMX]	Figure S3D
DB5789	pAde6^{PmeI}-Pdjc9-Djc9-(100-110)Δ-mCherry[hphMX]	Figure S3D
DB5790	pAde6^{PmeI}-Pdjc9-Djc9-(100-120)Δ-mCherry[hphMX]	Figure S3D
DB5791	pAde6^{PmeI}-Pdjc9-Djc9-L242A/I246A-mCherry[hphMX]	Figure S3D
DB5792	pAde6^{PmeI}-Pdjc9-Djc9-L261R-mCherry[hphMX]	Figure S3D
DB5793	pGEX4T-1-GST-Asf1	Figure 3F, 3H
DB5794	pCDFDuet-1-H3-L126A/L130A-FLAG$_2$-His$_6$–H4.1–H4.1	Figure 3F
DB5795	pETDut-His$_6$-GS-(HRV-3C)-Asf1	Figure 3G
DB5796	pDUAL-Pasf1-Asf1-YFP-FLAG-His$_6$	Figure 3H
DB5797	pDUAL-Pasf1-Asf1(1-161)-YFP-FLAG-His$_6$	Figure 3H
DB5798	pDUAL-Pasf1-Asf1-V95R-YFP-FLAG-His$_6$	Figure 3H
DB5799	pDUAL-Pinv1-H3-FLAG	Figure 5C
DB5800	paAde6^{NotI}-Pinv1-H3-FLAG[hphMX]	Figure 6D, S6F
DB5801	pade6^{NotI}-Pinv1-H3-K122E-FLAG[hphMX]	Figure S6F
DB5802	pDUAL-hhf2–hht2–FLAG	Figure 4B, 4C, 4D, S4B, 5D, S5C
DB5803	pDUAL-hhf2–hht2-K122E–FLAG	Figure 4A, 4C, S4B, 5D
DB5804	pDUAL-hhf2–hht2-K122R–FLAG	Figure 4C, S4A, S4B, 5D
DB5805	pDUAL-FLAG-hhf2–hht2	Figure S4C, 5E, S5B
DB5806	pDUAL-FLAG-hhf2–hht2-K122E	Figure S4D
DB5807	pDUAL-Pnmt1-H3-GFP	Figure 7A–7C
DB5808	pDUAL-Pnmt1-H3-FLAG	Figure 7D–7E
DB5809	pDUAL-Pnmt1-H3-Y41A-FLAG	Figure 7D–7E
DB5810	pDUAL-Pnmt1-H3-Y41E-FLAG	Figure 7D–7E
DB5811	pDUAL-Pnmt1-H3-L48A-FLAG	Figure 7D–7E
DB5812	pDUAL-Pnmt1-H3-L48R-FLAG	Figure 7D–7E
DB5813	pDUAL-Pnmt1-H3-I51A-FLAG	Figure 7D–7E
DB5814	pDUAL-Pnmt1-H3-Q55A-FLAG	Figure 7D–7E
DB5815	pDUAL-Pnmt1-H3-K122E-FLAG	Figure 7F–7G
DB5816	pDUAL-Pnmt1-H3-Y41A/K122E-FLAG	Figure 7F–7G
DB5817	pDUAL-Pnmt1-H3-Y41E/K122E-FLAG	Figure 7F–7G
DB5818	pDUAL-Pnmt1-H3-L48A/K122E-FLAG	Figure 7F–7G
DB5819	pDUAL-Pnmt1-H3-I51A/K122E-FLAG	Figure 7F–7G
DB5820	pDUAL-Pnmt1-H3-Q55A/K122E-FLAG	Figure 7F–7G
